# Supplementary material for: IGF1R/IR Mediates Resistance to BRAF and MEK Inhibitors in BRAF-Mutant Melanoma
Source: Cancers (Basel). 2021 Nov 22;13(22):5863. doi: 10.3390/cancers13225863 (PMC8616282; doi:10.3390/cancers13225863)
Supplement: Supplementary file 1 [file cancers-13-05863-s001.zip › cancers-1433924 suppl.pdf]

Supplementary Files

IGF1R/IR Mediates Resistance to BRAF and MEK Inhibitors in BRAF-Mutant Melanoma

Hima Patel, Rosalin Mishra, Nour Yacoub, Samar Alanazi, Mary Kate Kilroy and Joan T. Garrett

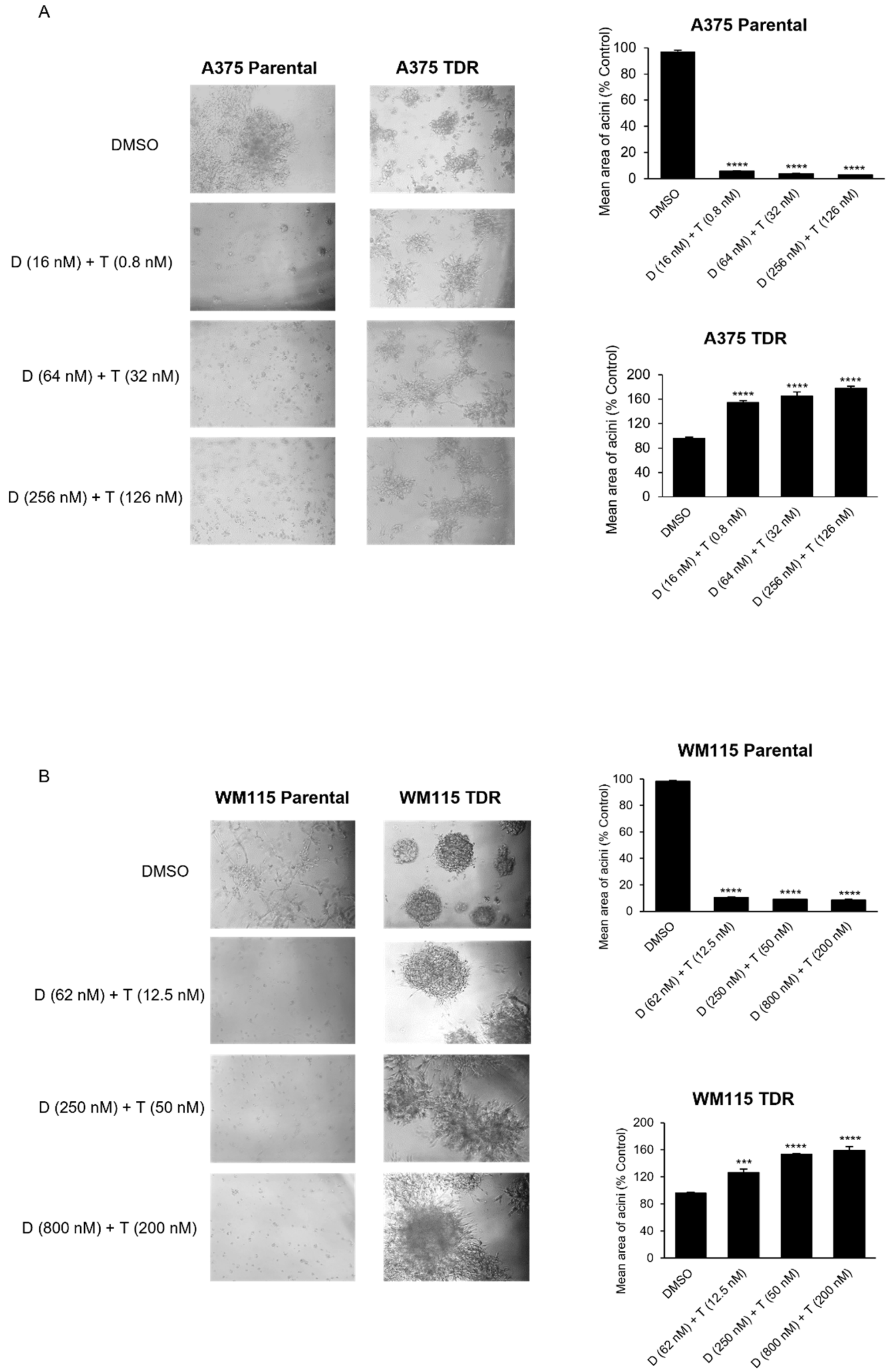

Figure S1. Dabrafenib and trametinib inhibit growth of parental cells but not TDR cells. (**A**) A375 and (**B**) WM115 Parental and TDR cells (2 x 104 cells/well) were seeded on matrigel basement membrane and treated with indicated drugs every second day. 5 pictures of different areas of each well were captured on day 21 (left panel) at 10× magnification. Quantification of mean area of acini (Right panel).*** p <0.001; **** p <0.001 by two-tailed Student's t-test compared to DMSO group.

A

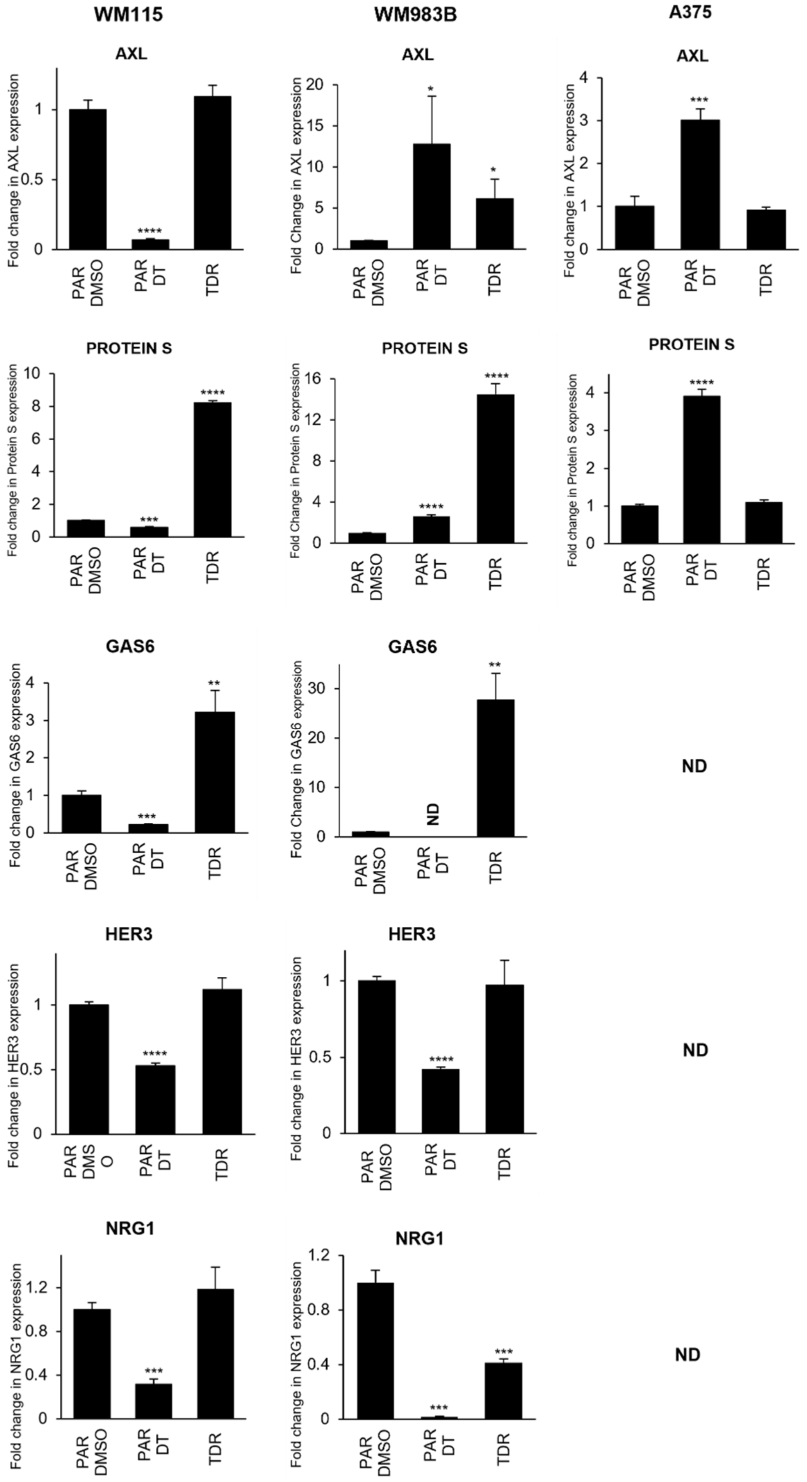
WM115
WM983B
A375
AXL
Fold change in AXL expression
Fold Change in AXL expression
PROTEIN S
Fold change in Protein S expression
Fold Change in Protein S expression
GAS6
Fold change in GAS6 expression
Fold Change in GAS6 expression
HER3
Fold change in HER3 expression
NRG1
Fold change in NRG1 expression
PAR DMSO
PAR DT
TDR
ND
*
**
***
****

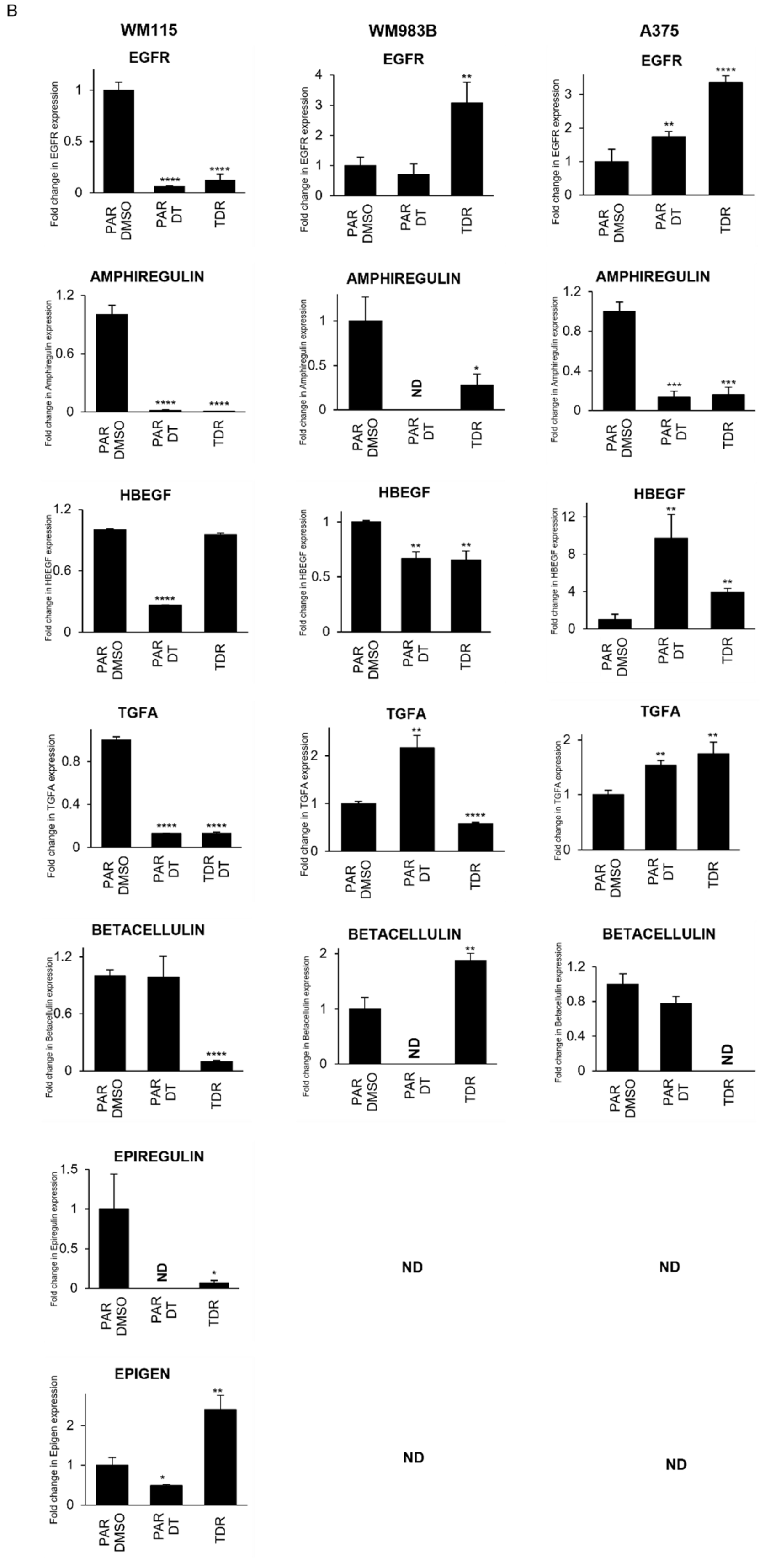

Figure S2. mRNA expression of AXL, HER3, EGFR and their ligands in parental and TDR cells. Quantification of Actin- normalized mRNA levels using real-time quantitative PCR for (**A**) AXL, PROTEIN S, GAS6, HER3, NRG1 and (**B**) EGFR, AMPHIREGULIN, HBEGF, TGFA, BETACELLULIN, EPPIREGULIN, EPIGEN for parental cells treated with DMSO or dabrafenib

(D) + trametinib (T) (WM115: D: 800 nM, T: 200 nM; WM983B: D: 2.4 μM, T: 500 nM; A375: D: 250 nM, T: 12.5 nM) and TDR following 24 hours of treatment. * $p < 0.05$; ** $p < 0.01$; *** $p < 0.001$; **** $p < 0.0001$ by two-tailed Student's *t*-test. ND: Not detectable.

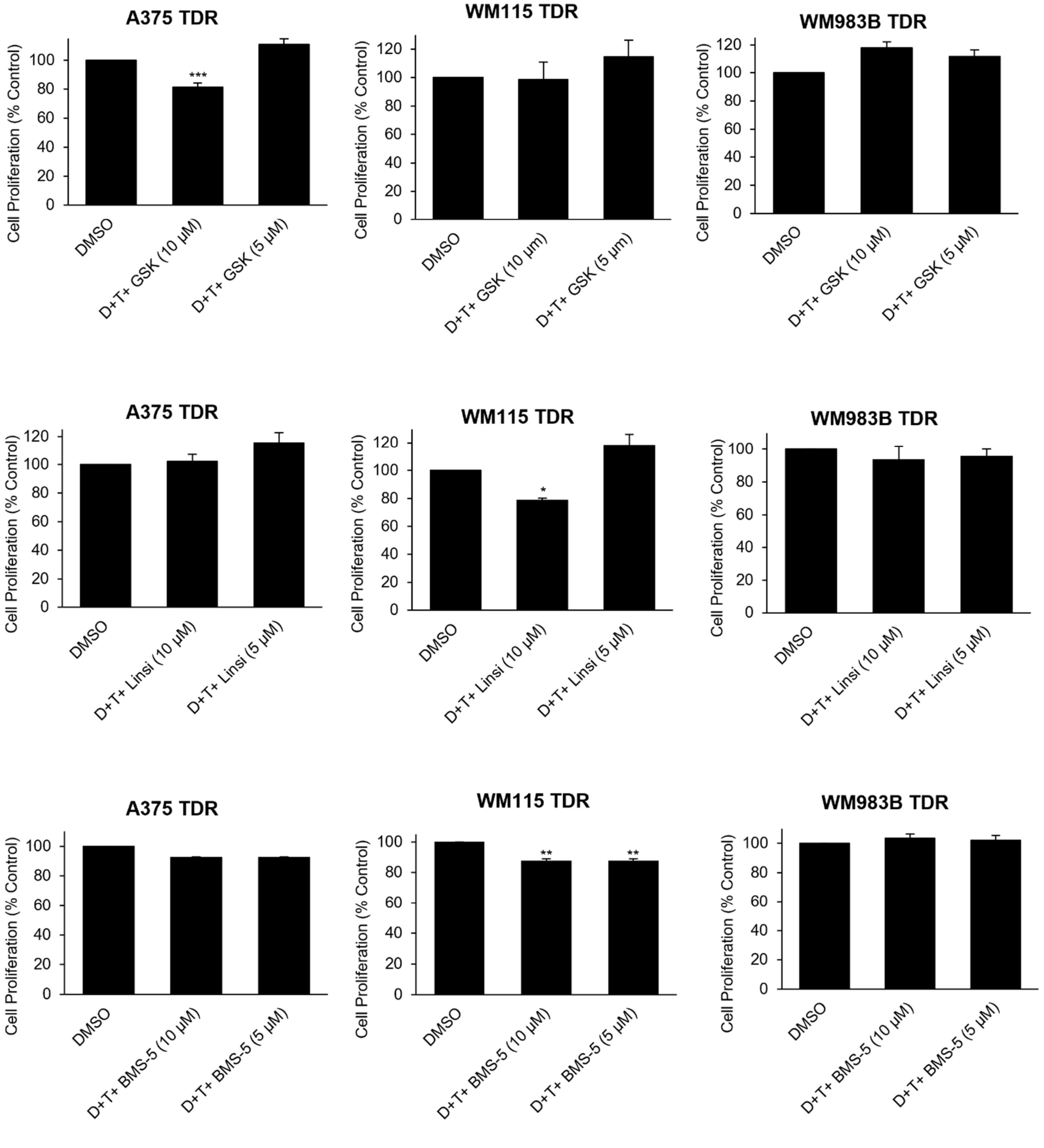

Figure S3. Effect of Linsitinib, BMS-536924 and GSK1838705A on TDR cell proliferation.TDR cells (WM115, WM983B and A375; 1 × 104 cells/well) plated for 24 h and treated with DMSO, dabrafenib (D) and trametinib (T) (WM115: D: 800 nM, T: 200 nM; WM983B: D: 2.4 μM, T: 500 nM; A375: D: 250 nM, T: 12.5 nM) in combination with Linsitinib (Linsi), BMS-536924 (BMS-5) and GSK1838705A (GSK) (5 μM and 10 μM) and treated with MTT. The intensities are represented as mean, Error bars: SEM * $p < 0.05$, ** $p < 0.01$, *** $p < 0.001$ by two-tailed Student's *t*-test compared to DMSO group.

A

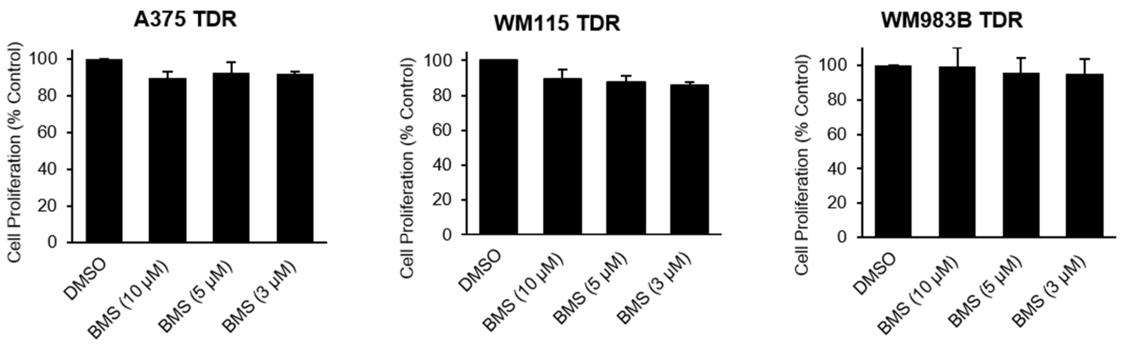

B

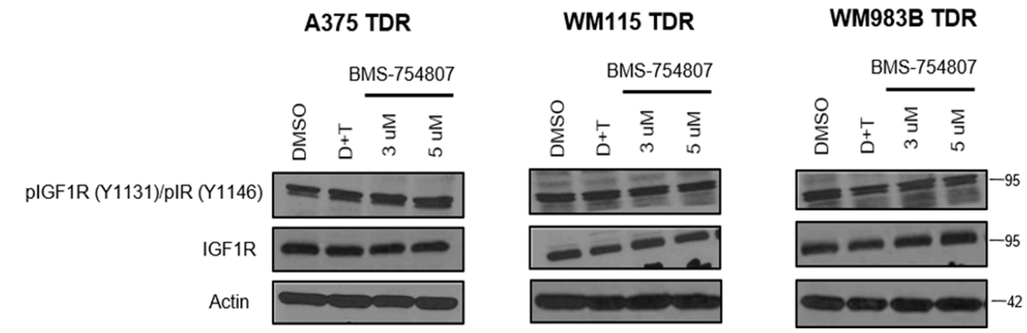

Figure S4. BMS-754807 does not inhibit proliferation and p-IGF1R/p-IR expression in TDR cells. (**A**) TDR cells (WM115, WM983B and A375) were plated and treated with DMSO, BMS-754807 (BMS; 10 µM, 5 µM or 3 µM) for 72 h. Cells were treated with MTT for 4 h and absorbance was read at 570 nm. Data are presented as mean ± SEM. (**B**) Immunoblots for A375, WM115 and WM983B TDR cells treated with DMSO, dabrafenib (D) and trametinib (T) (WM115 TDR: D: 800 nM, T: 200 nM; WM983B TDR: D: 2.4 µM, T: 500 nM; A375 TDR: D: 250 nM, T: 12.5 nM), or BMS-754807 (5 µM or 3 µM) for 24 h. Whole cell lysates were analyzed for western blot using indicated antibodies. Actin used as loading control.

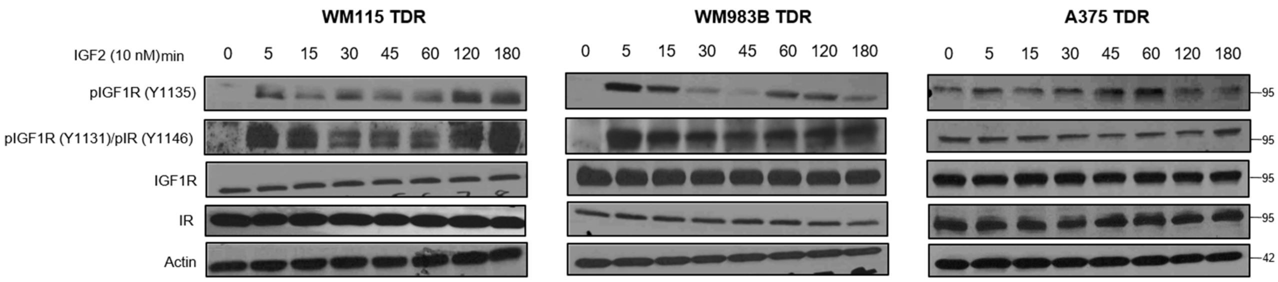

Figure S5. IGF2 dependent activation of IGF1R and IR in TDR cells. TDR cells were serum starved for 24 h and treated with IGF2 (10 nM) for indicated time. Whole cell lysates were analyzed for western blot using indicated antibodies. Actin used as loading control.

WM983B parental: p-ERK

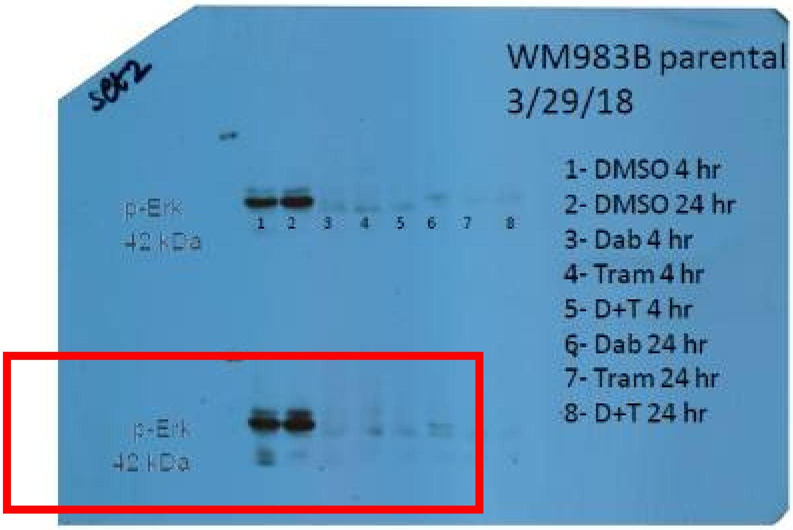

Figure 1D

WM983B parental - ERK

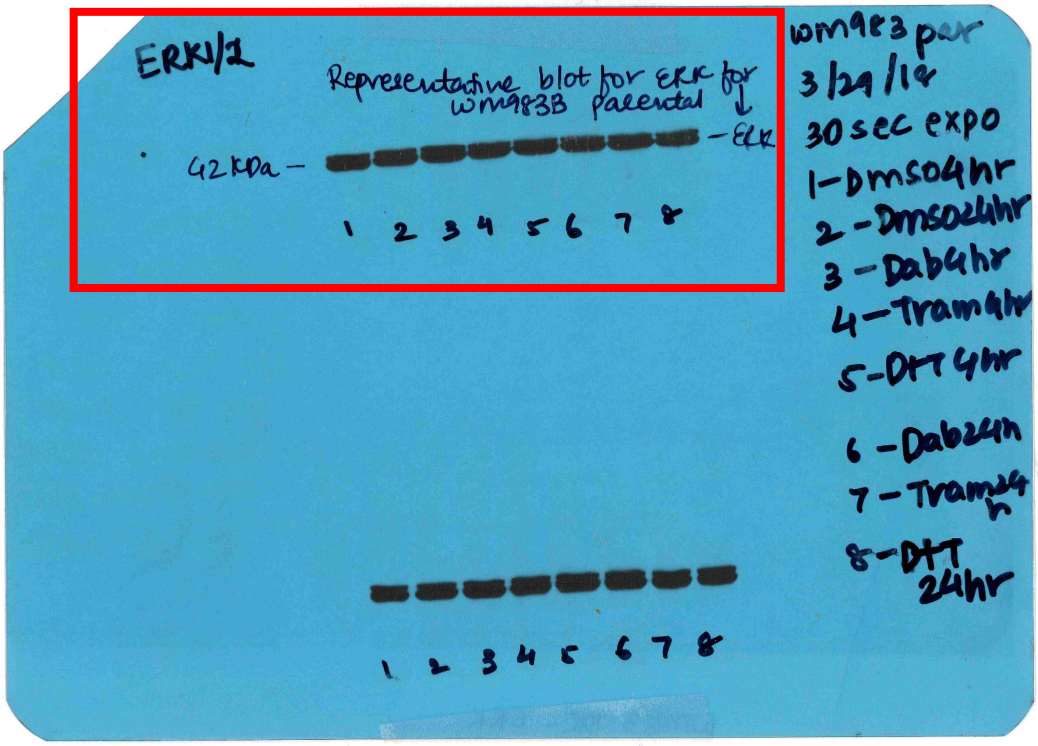

WM983B parental- Actin

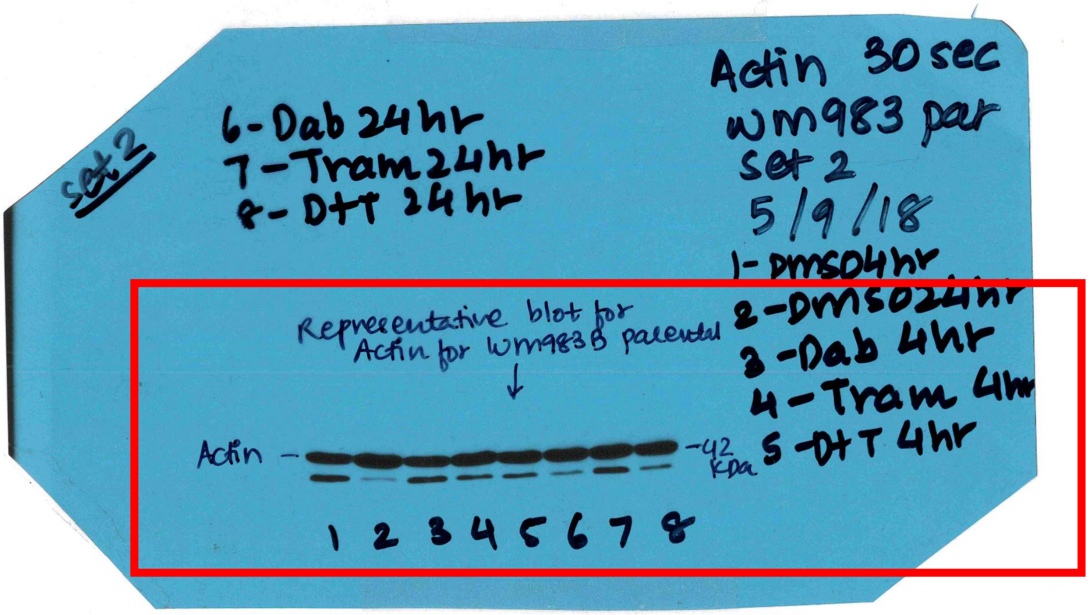

WM983 TDR - p-Erk

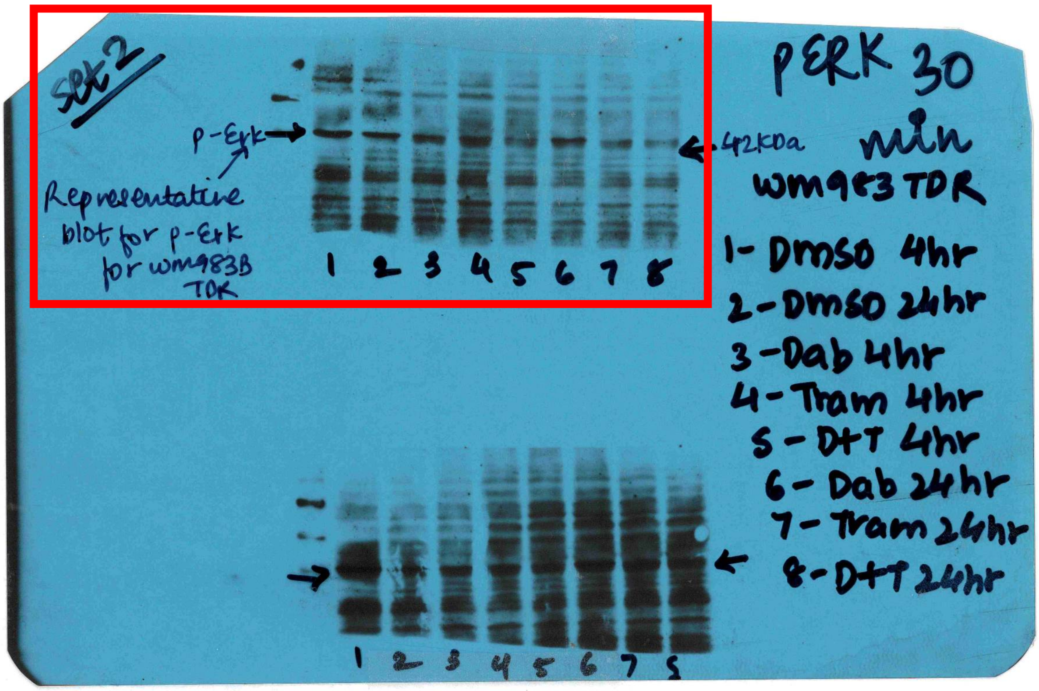

WM983 TDR - ERK

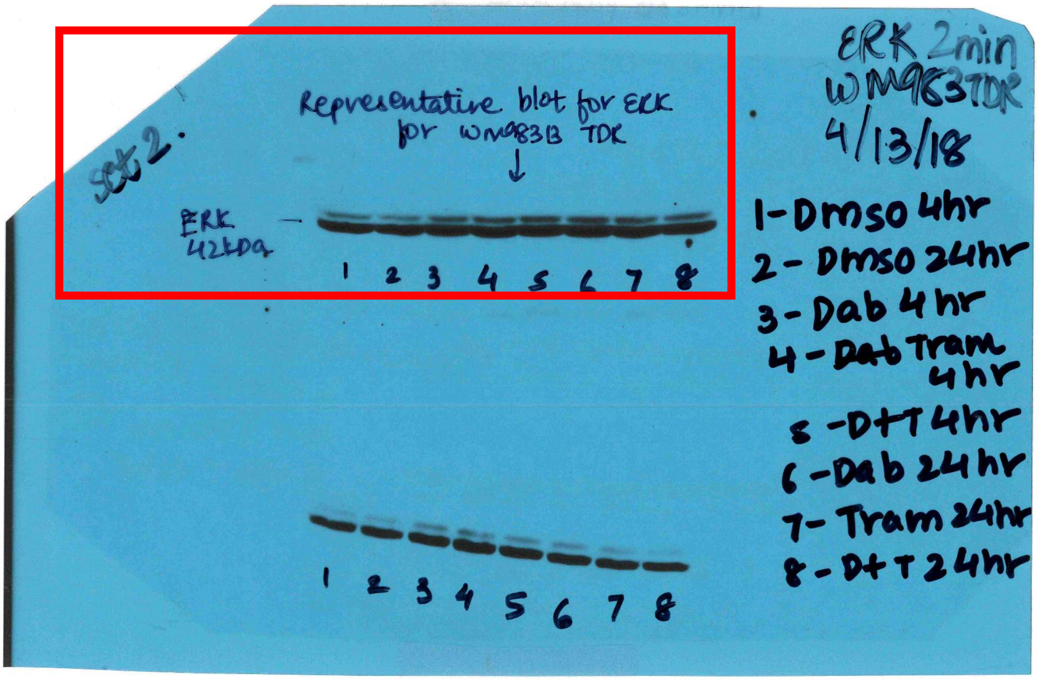

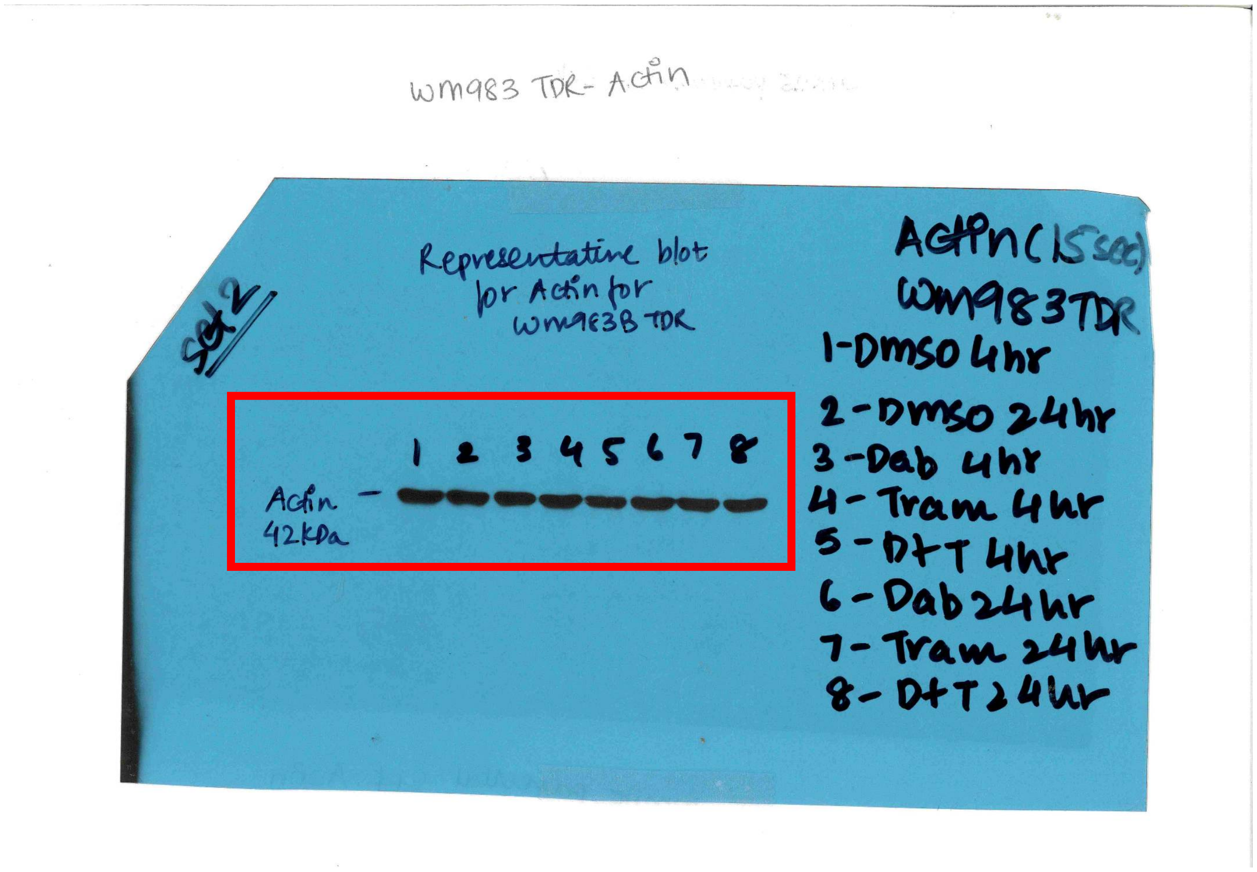
wm983 TDR- Actin
Representative blot for Actin for wm983B TDR
Set 2
Actin (15 sec)
wm983TDR
1-DMSO 4hr
2-DMSO 24hr
3-Dab 4hr
4-Tram 4hr
5-DTT 4hr
6-Dab 24hr
7-Tram 24hr
8-DTT 24hr
1 2 3 4 5 6 7 8
Actin
42KDa

WM115 parental p-Erk

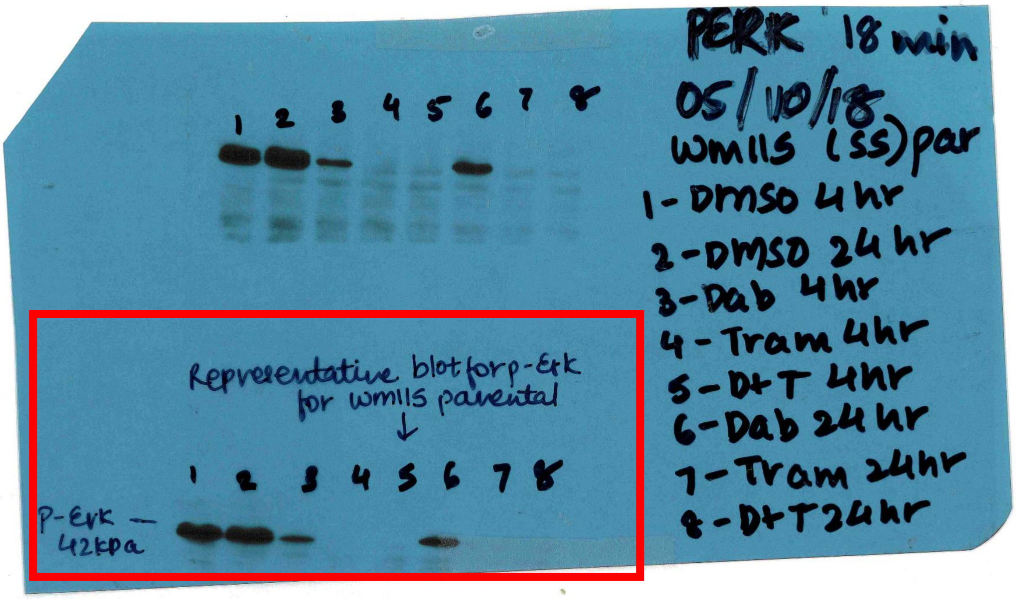

WM115 parental - ERK, Actin

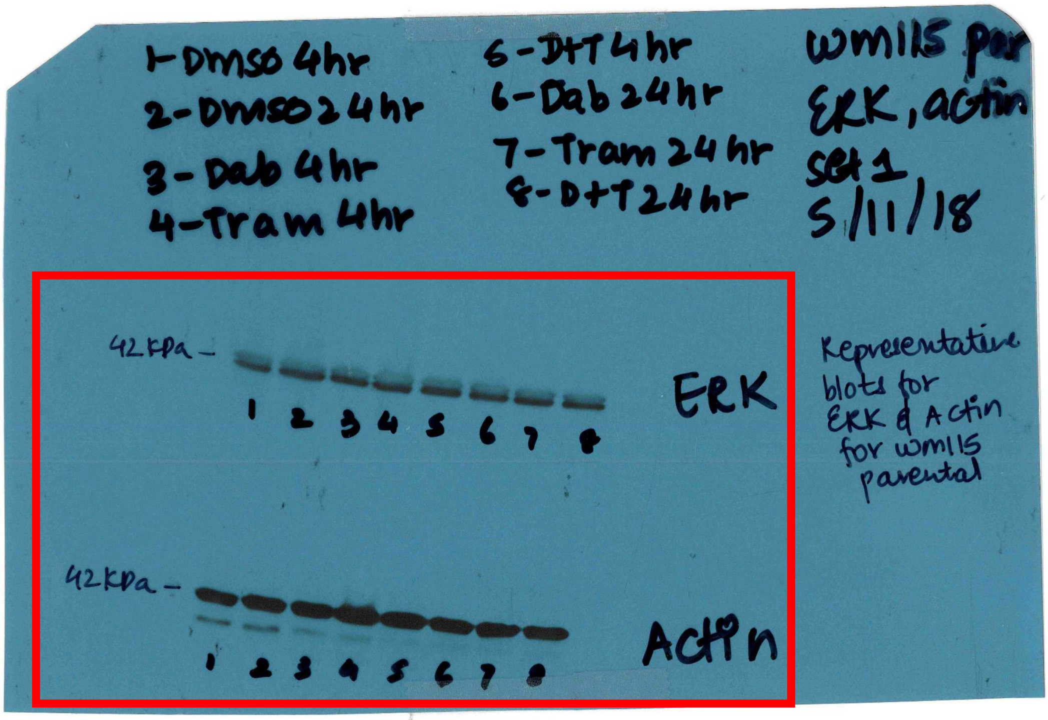

WM115TDR : p-Erk

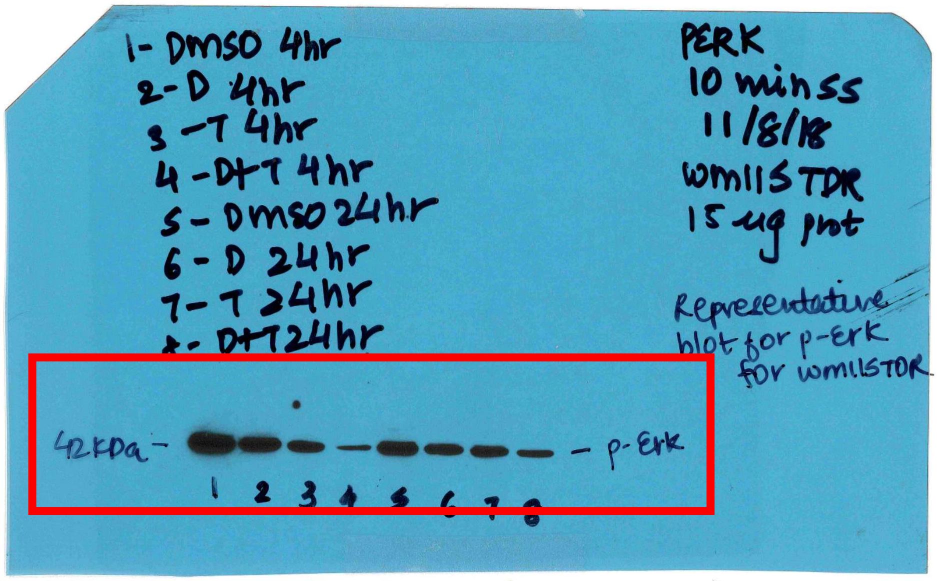

WM115 TDR: ERK

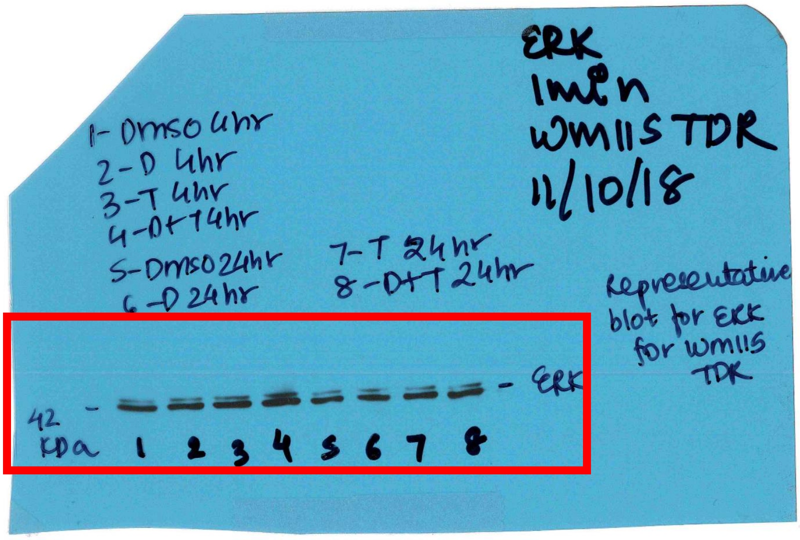

WM115TDR: Actin

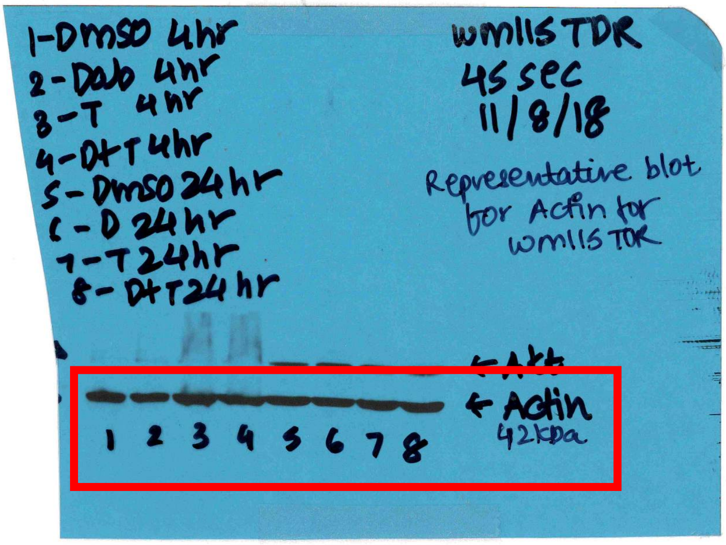

A375 parental: p-ERK

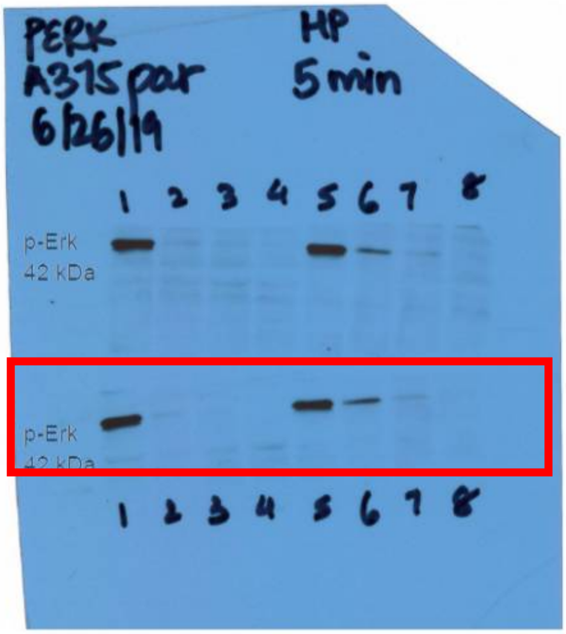

1 – DMSO 4 hr
2- Dab 4 hr
3- Tram 4 hr
4- D+T 4 hr
5- DMSO 24 hr
6- Dab 24 hr
7- Tram 24 hr
8- D+T 24 hr

A375 parental : ERK

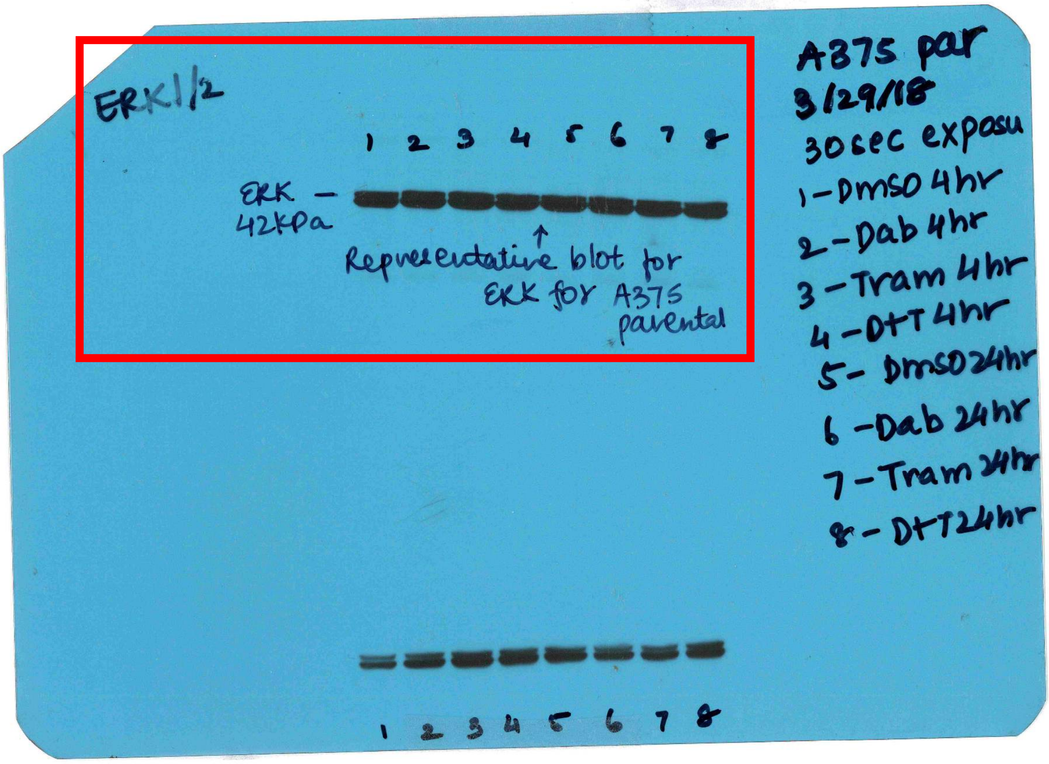

A375 parental : Actin

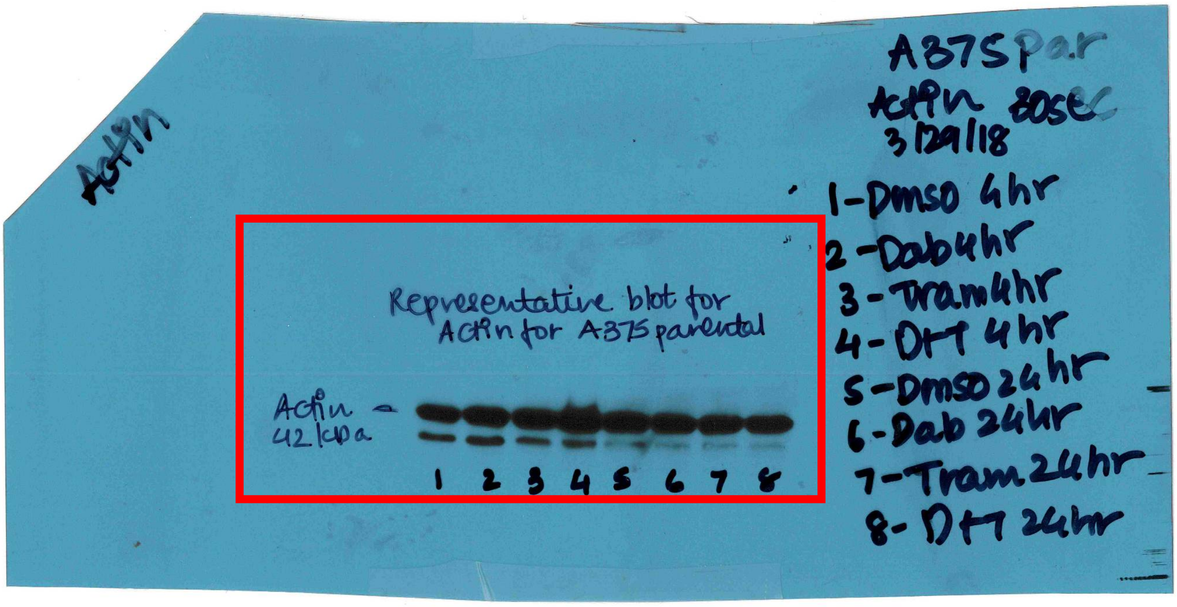

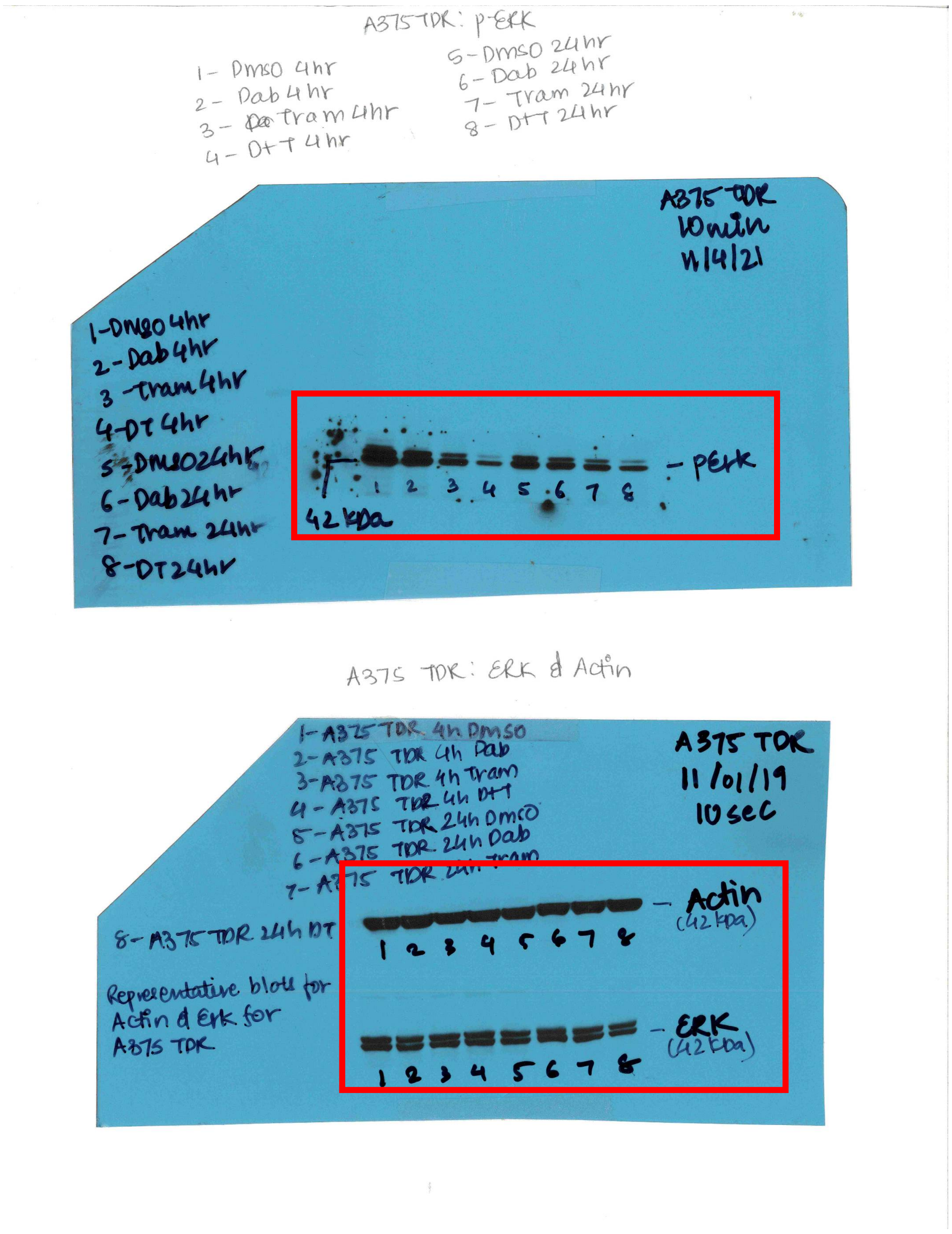

Figure S6. Whole blots for western blots in Figure 1D.

WM983BTDR: p-Erk

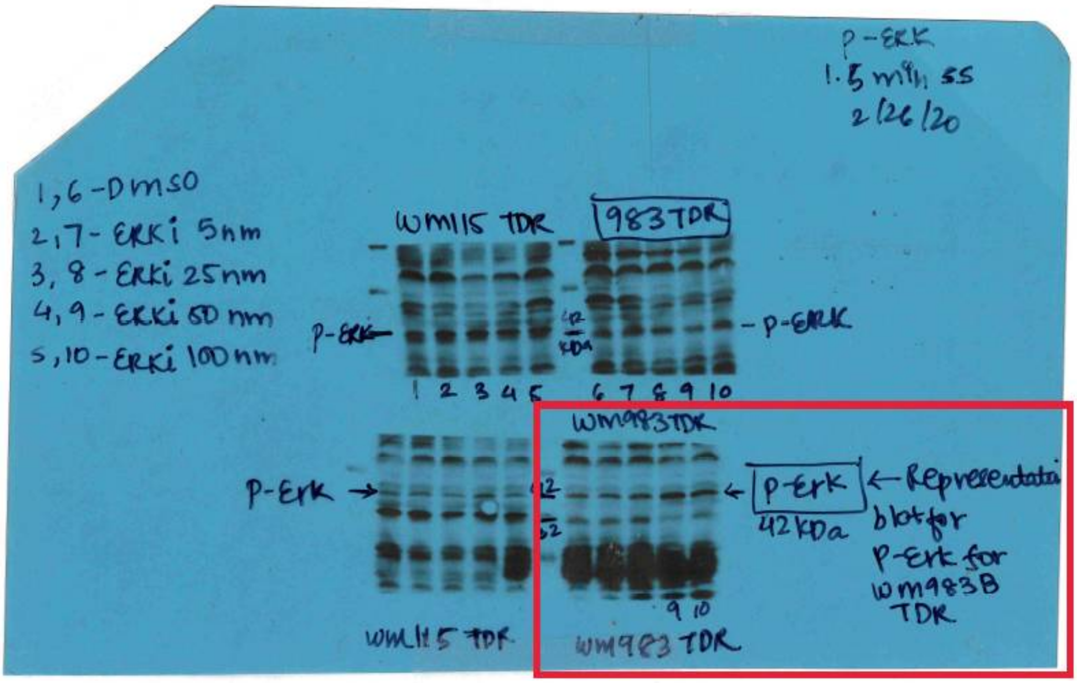

WM983B TDR: ERK & Actin

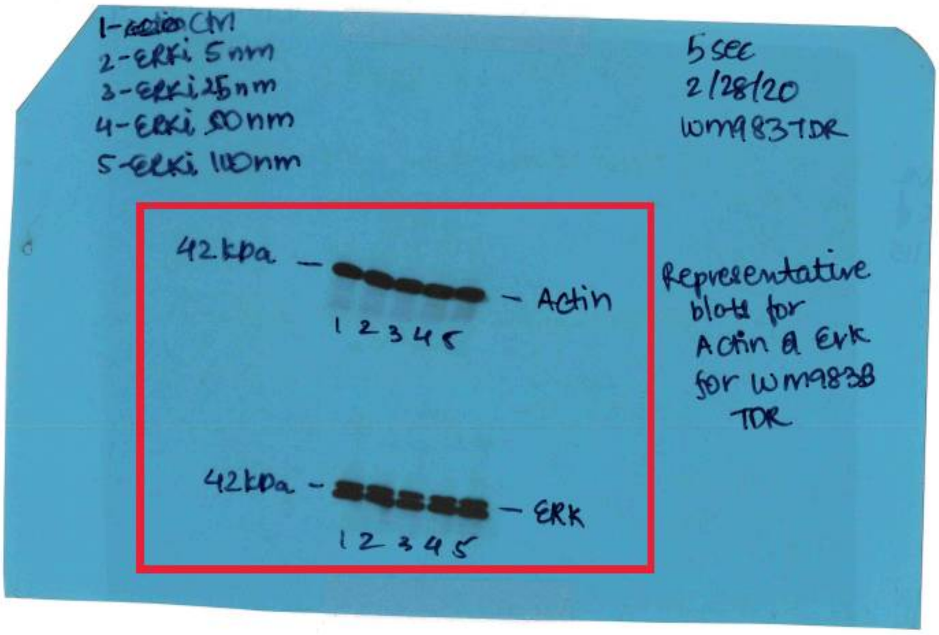

WM115TDR: p-Erk

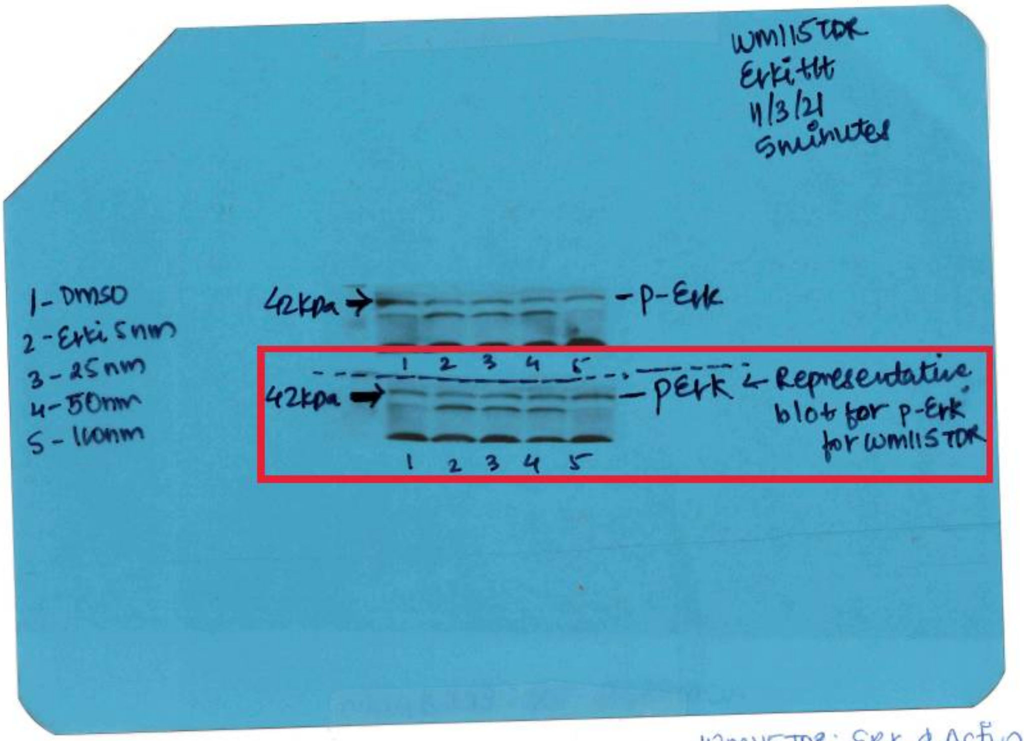

WM115TDR: ERK & Actin

Representative blots for ERK & Actin for WM115 TDR

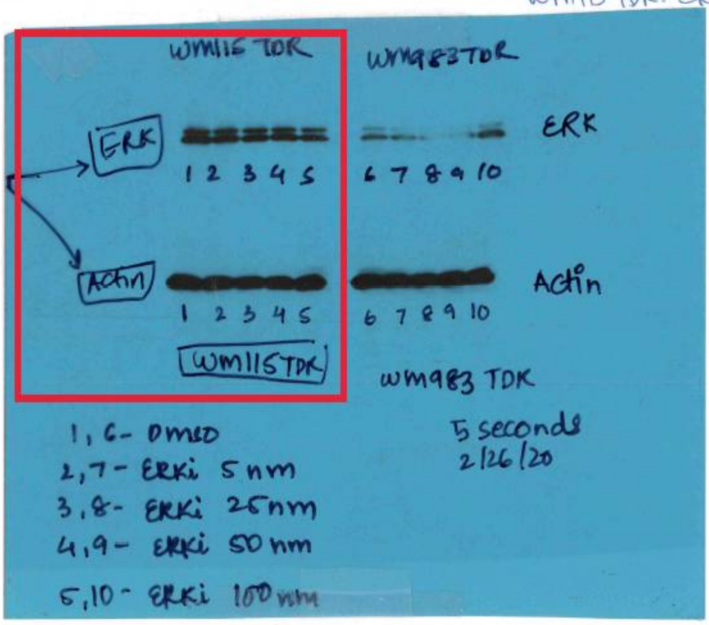

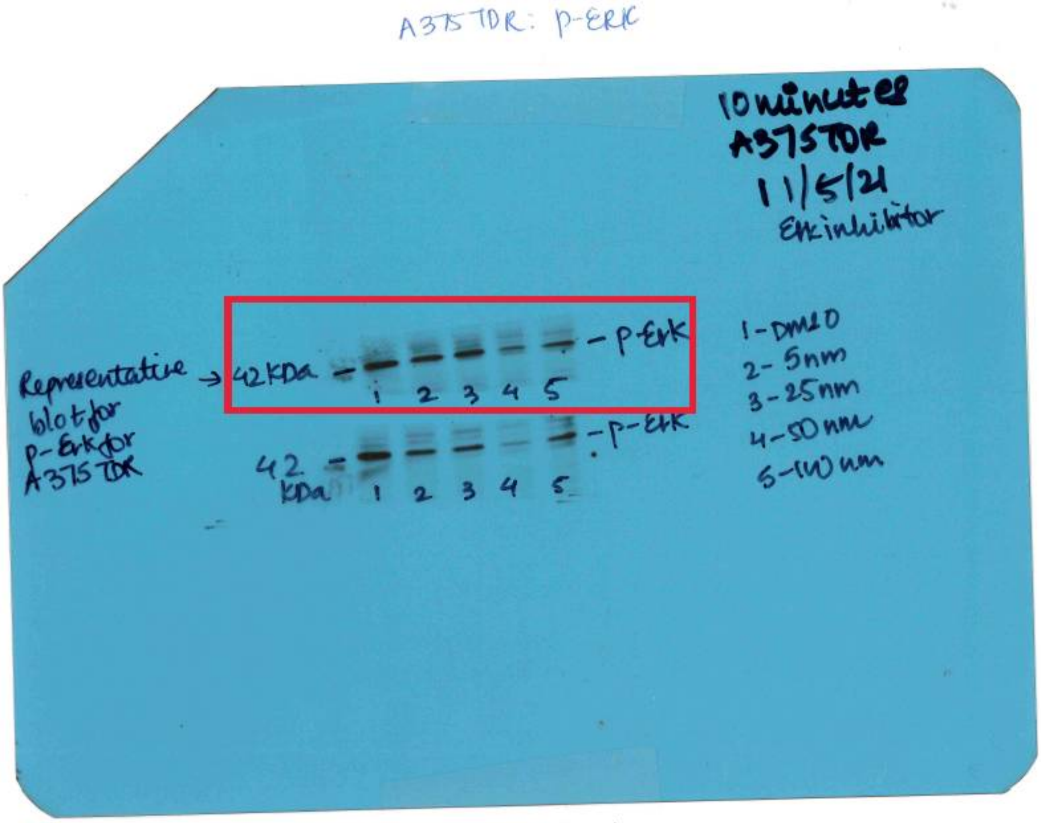

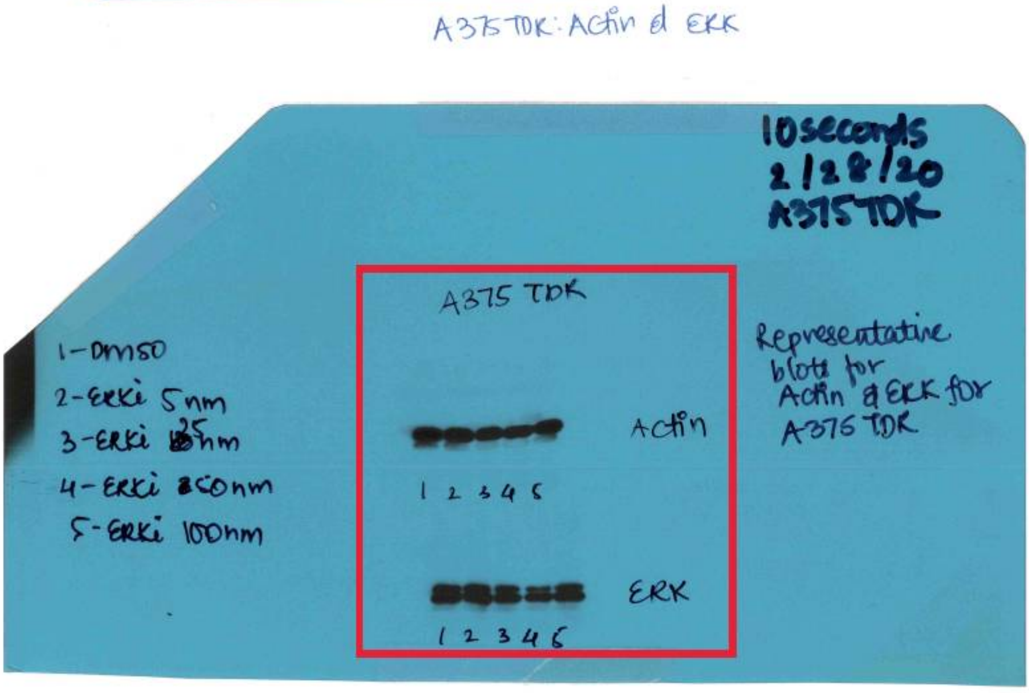

Figure S7. Whole blots for western blots in Figure 1E.

wm115: p-HER3(Y1289) & p-HER3(Y1197)
wm983: p-HER3 (Y1289) & p-HER3(Y1197)

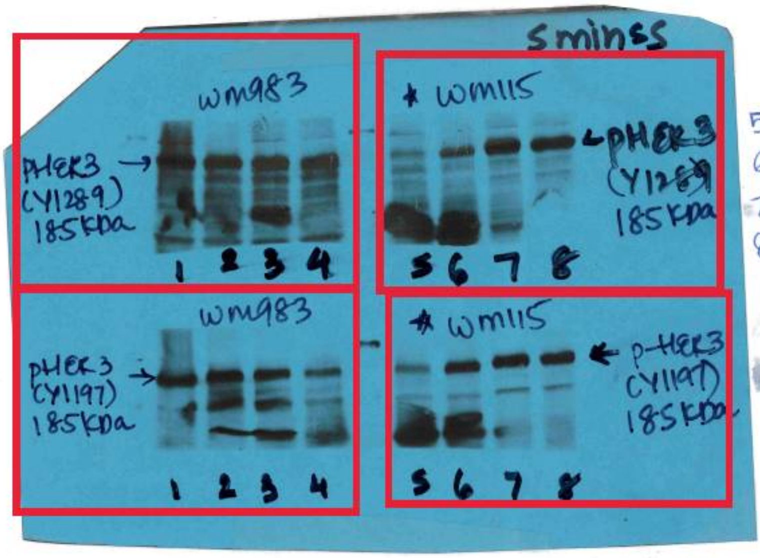

5- wm115pal DMSO
6- wm115 pal DT
7- wm115 TDR DMSO
8- wm115 TDR DT

* Representative blots for pHER3 (Y1289) & pHER3(Y1197) for wm115. ; pHER3 (Y1289) for wm983 pHER3 (Y11897)

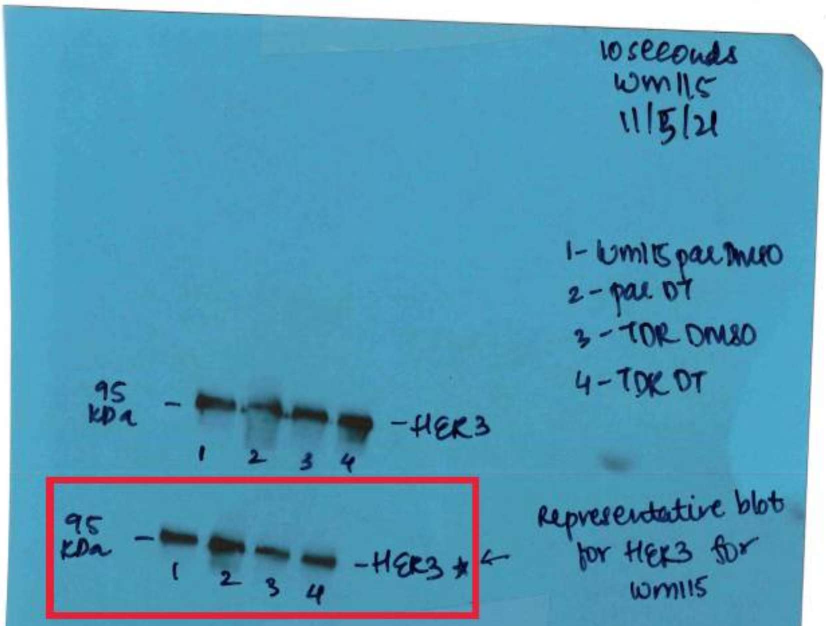

wm115: HER3

* Representative blot for HER3 for wm115

WM115: p-EGFR & EGFR

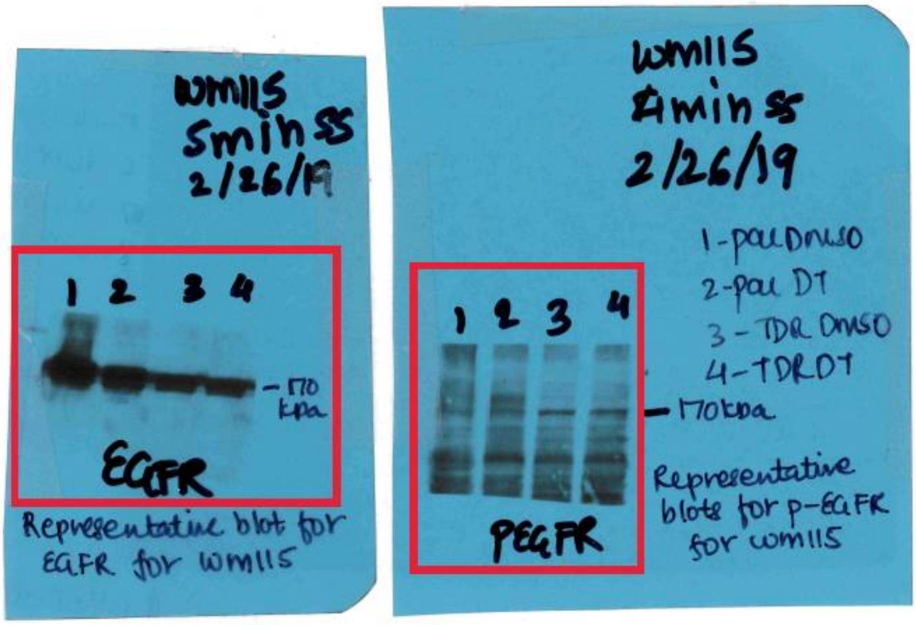

WM115: p-EGFR (Y1131) / p-IR (Y1146)

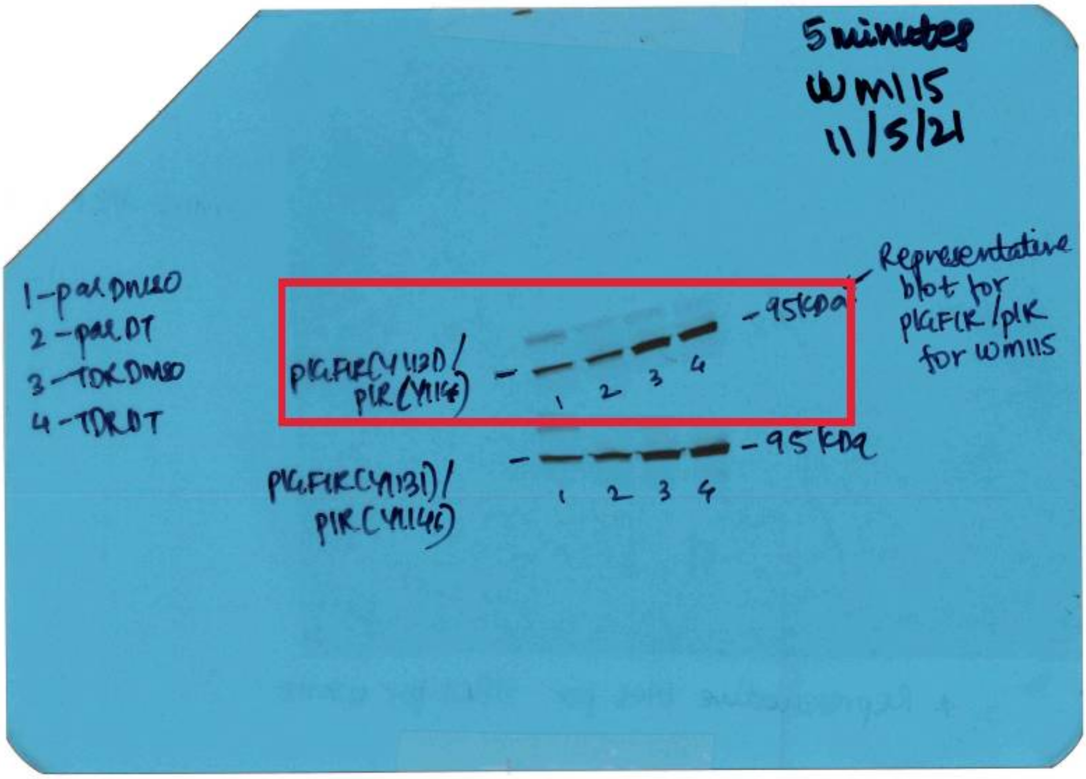

wm983B: Axl; IGFIR   wm115: Axl; IGFIR

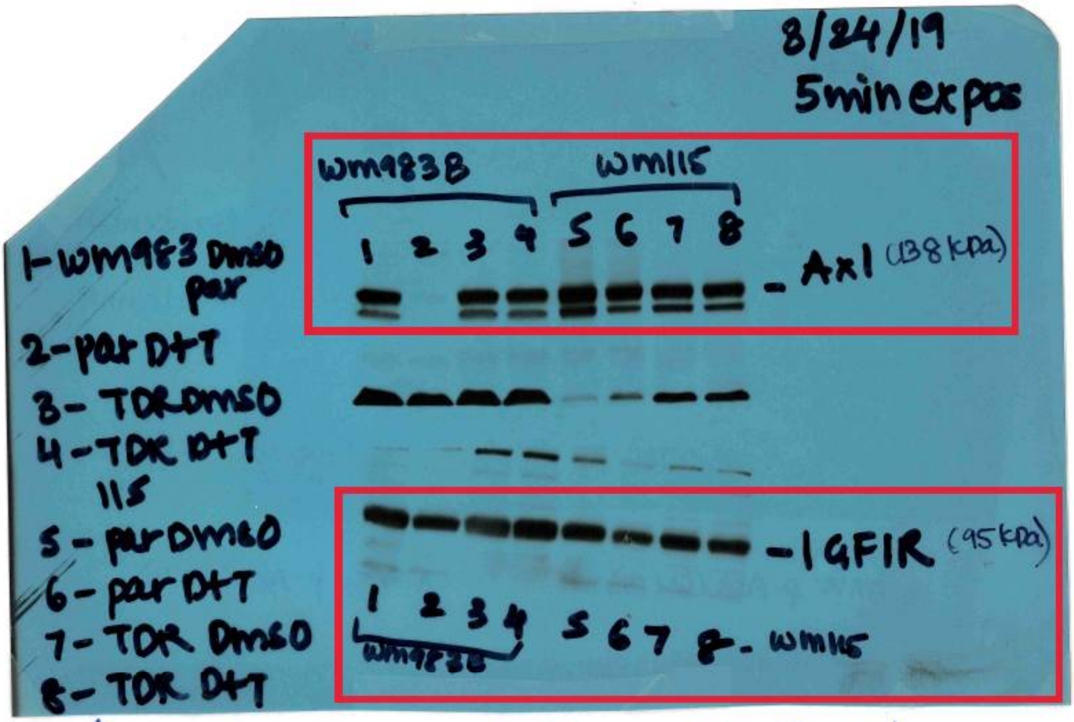

↑
Representative blots for wm983B (Axl & IGFIR) and wm115 (Axl & IGFIR)

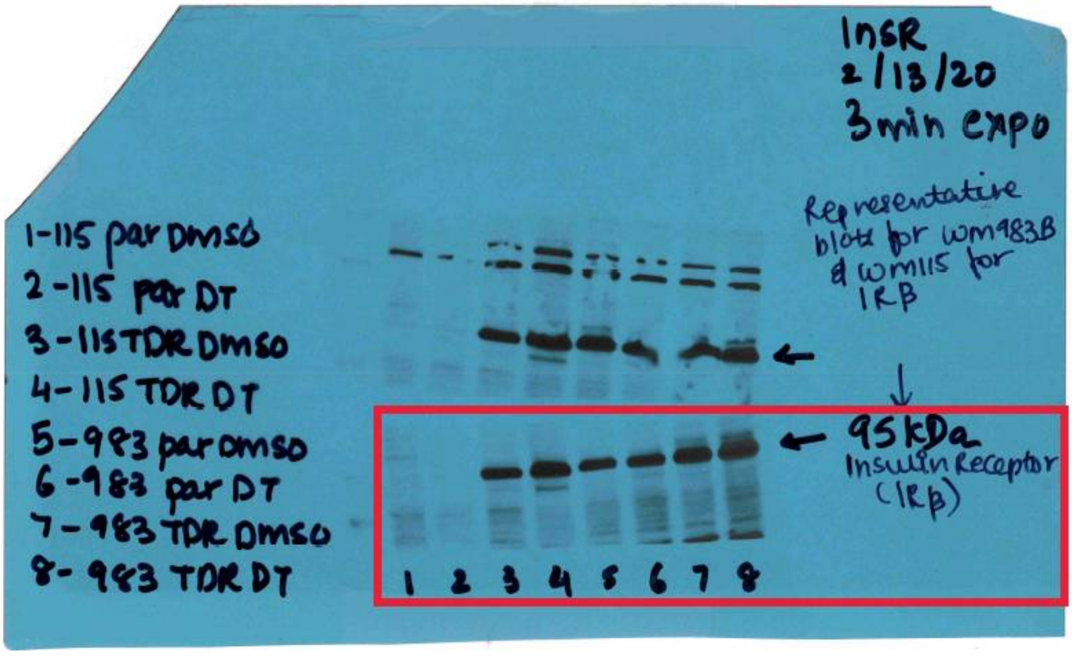

WM115: p-AXL (Y779)

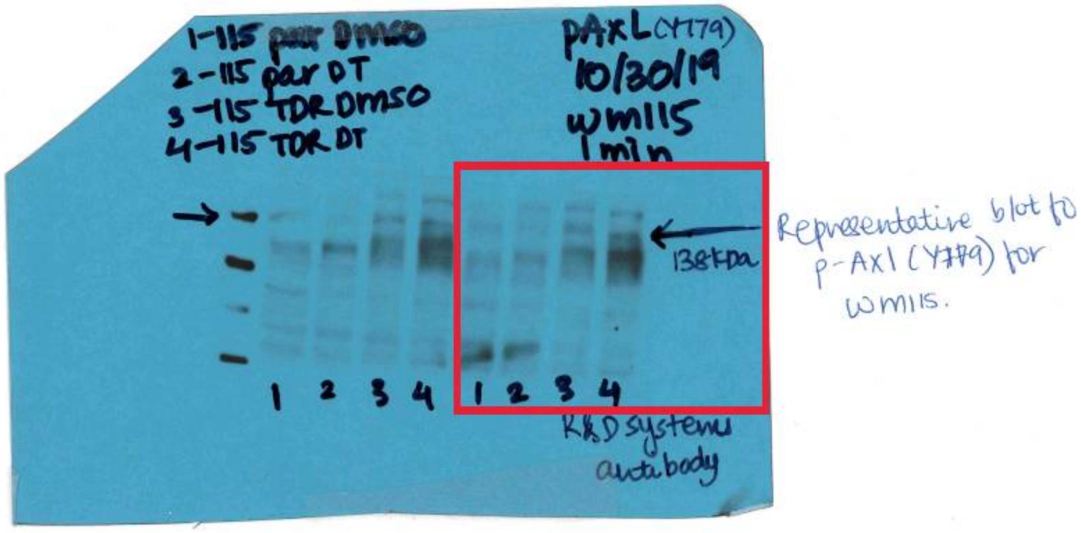

WM115: p-Akt (S473)

A 375: p-Akt (S473)

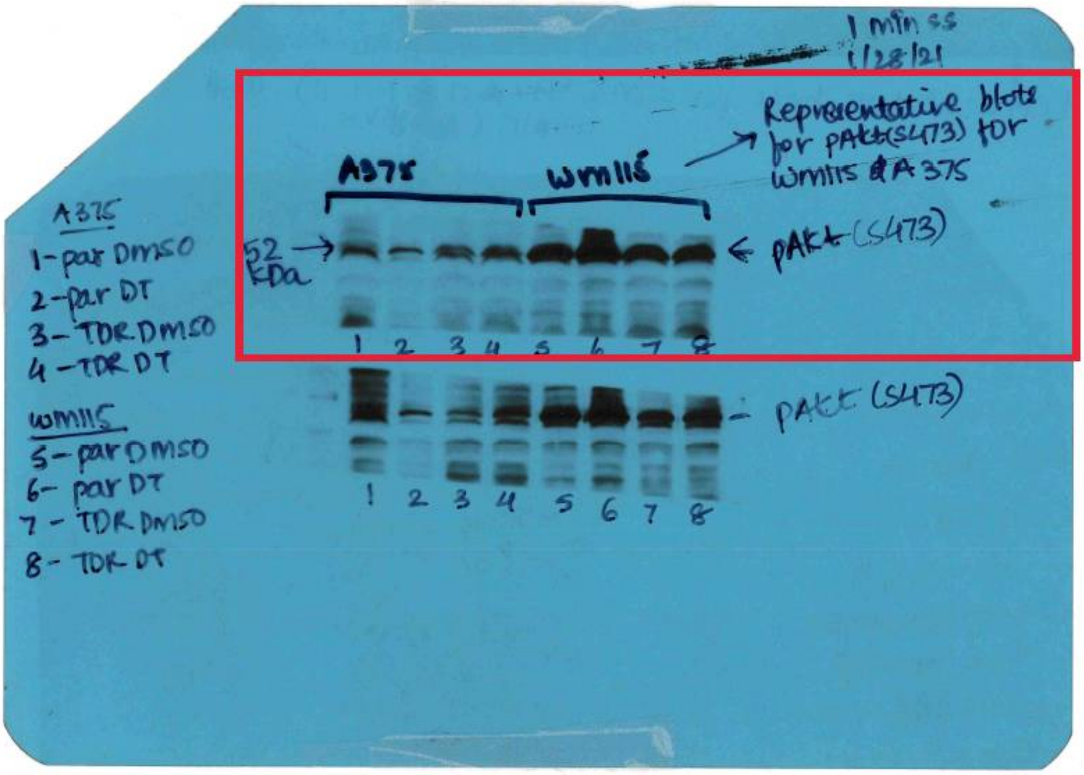

WM115: Akt ; A375: Akt

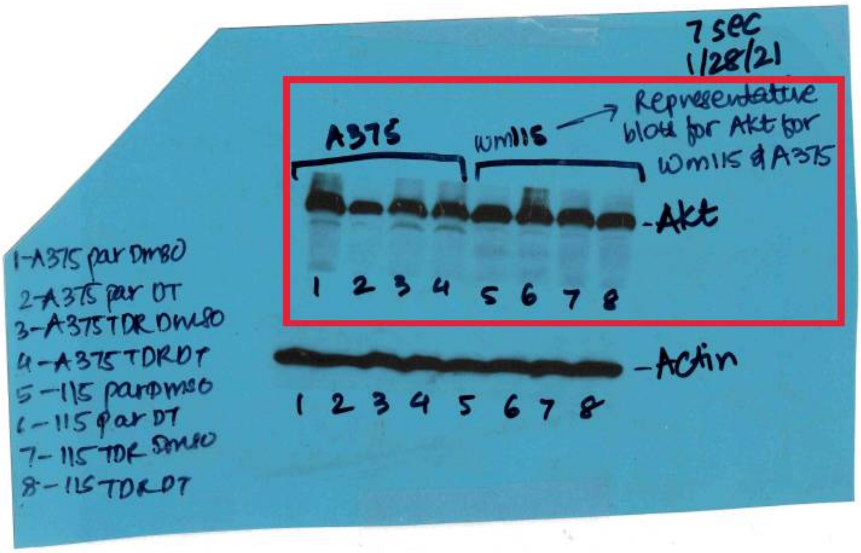

WM115: Actin

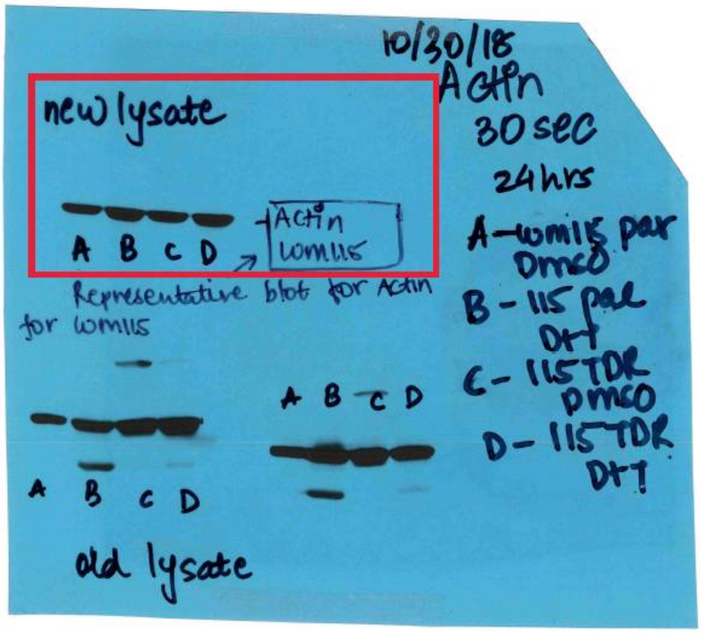

WM983B: HER3

*Representative blot for HER3 for WM983B

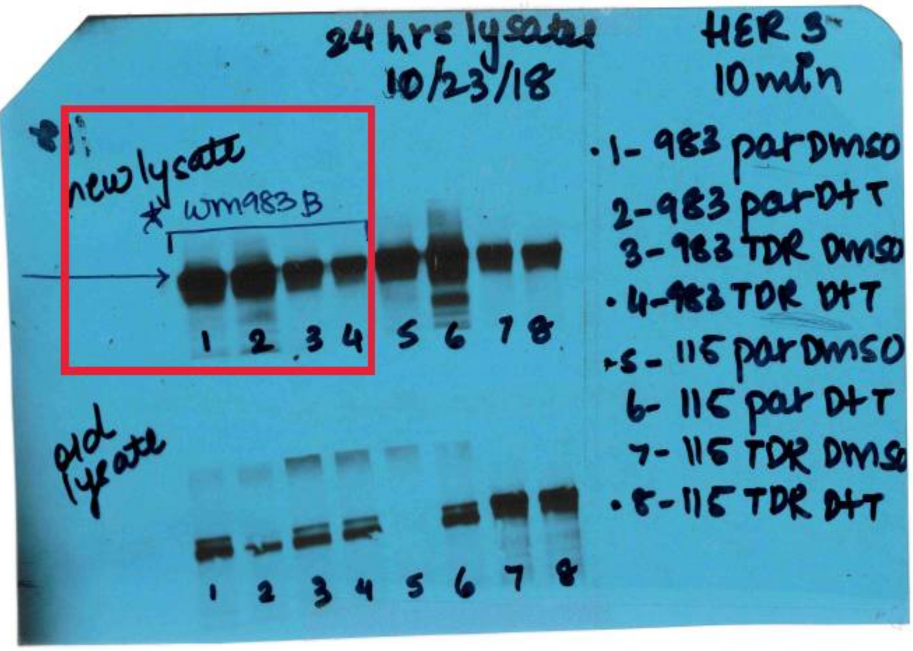

WM983B: p-EGFR & EGFR

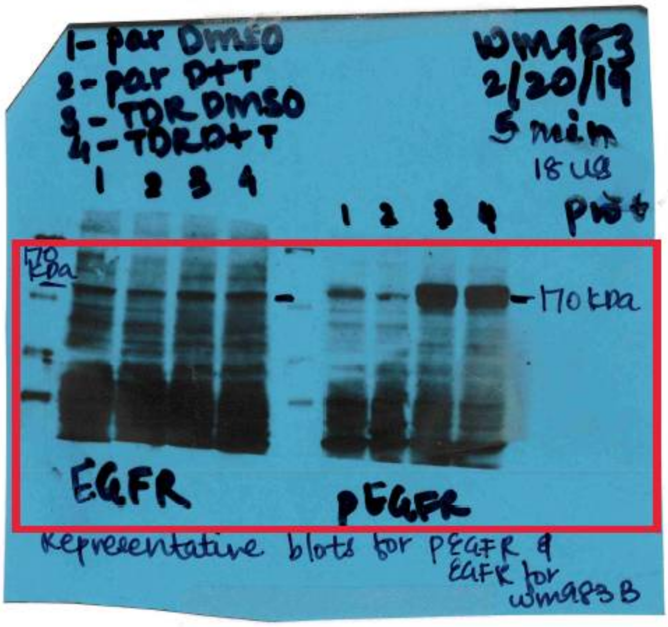

WM983B : P-AXL(Y779)

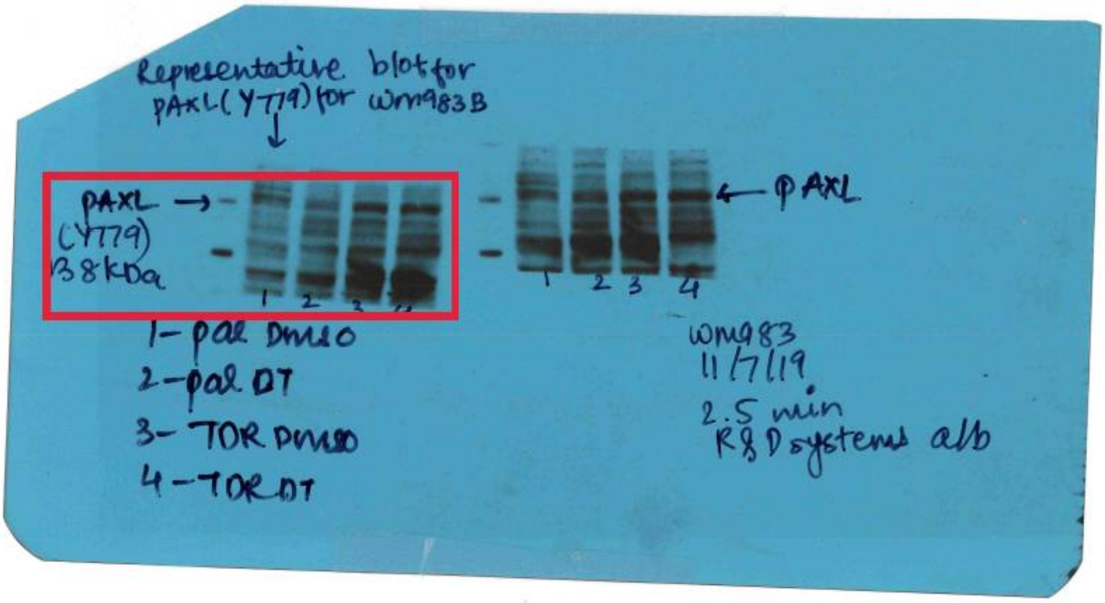

WM983B: p-Akt (S473)

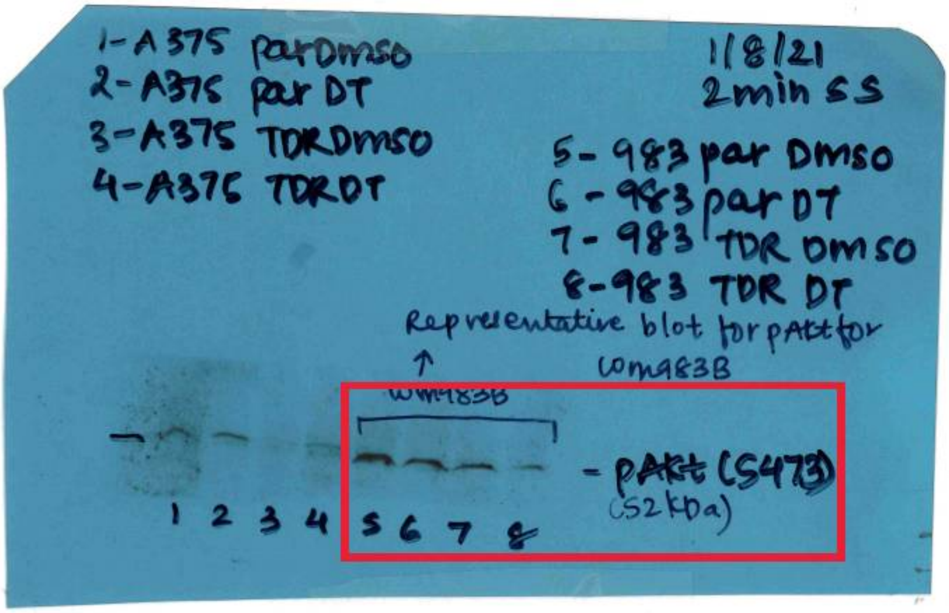

WM983B: Akt

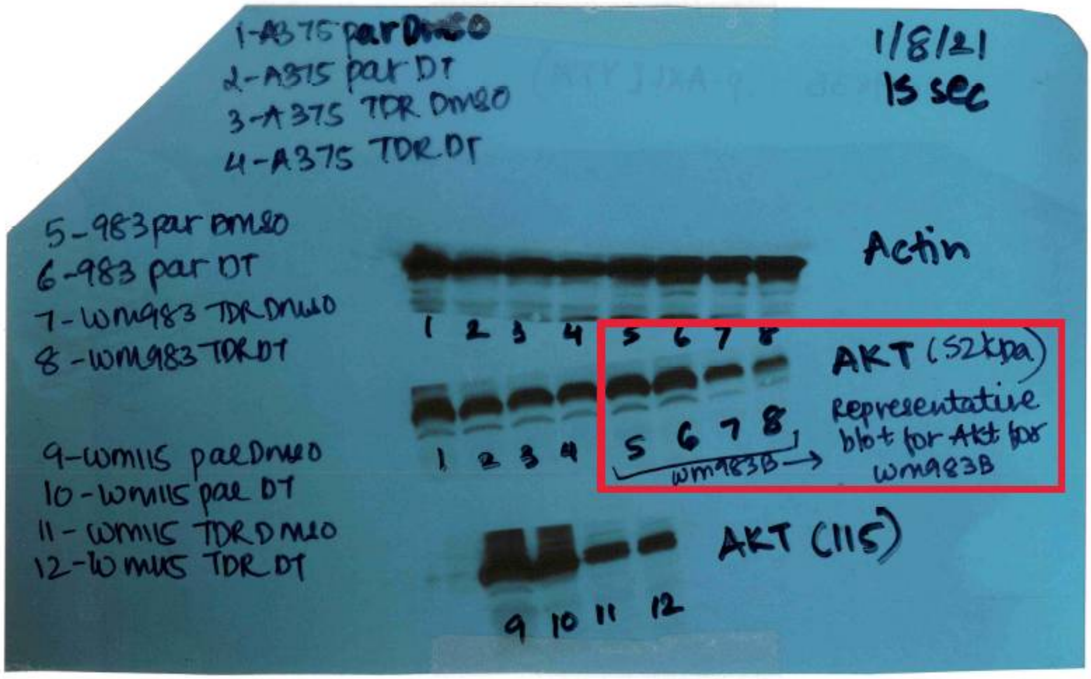

WM983B: Actin

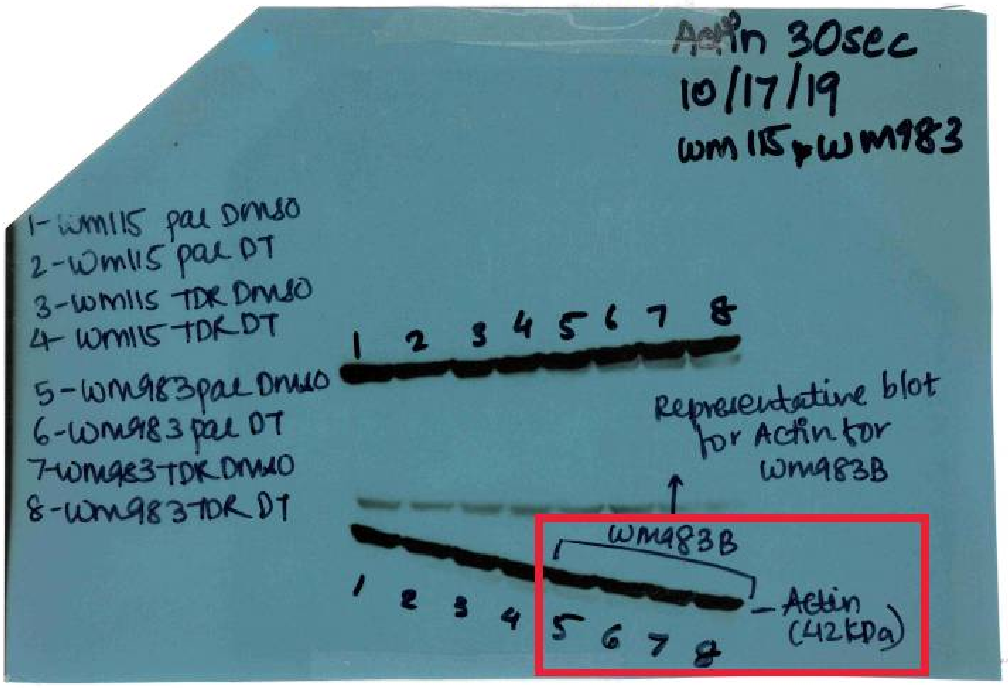

A375: p-HER3 (Y1289)

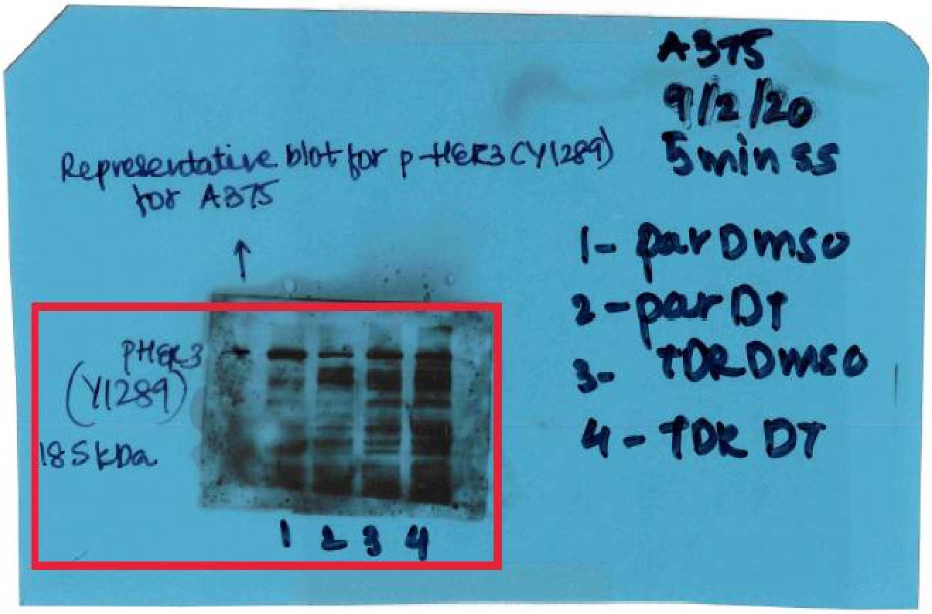

A375: p-HER3 (Y1197)

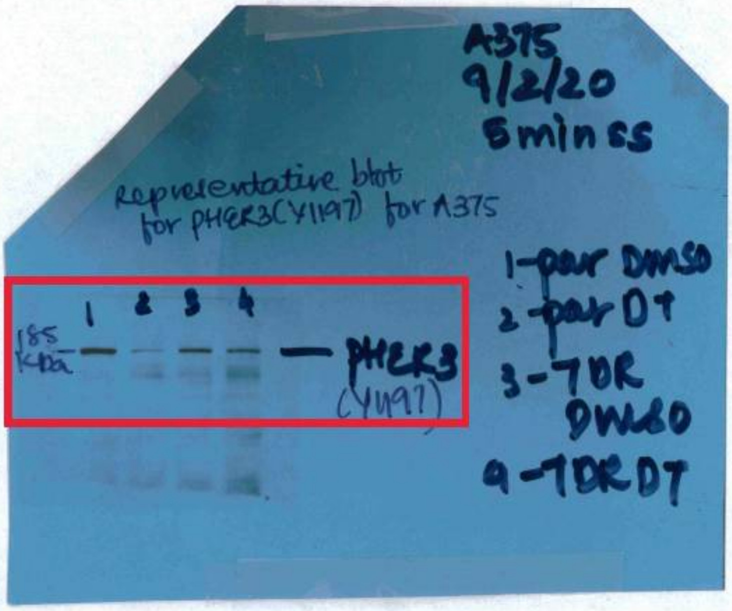

A375: HER3

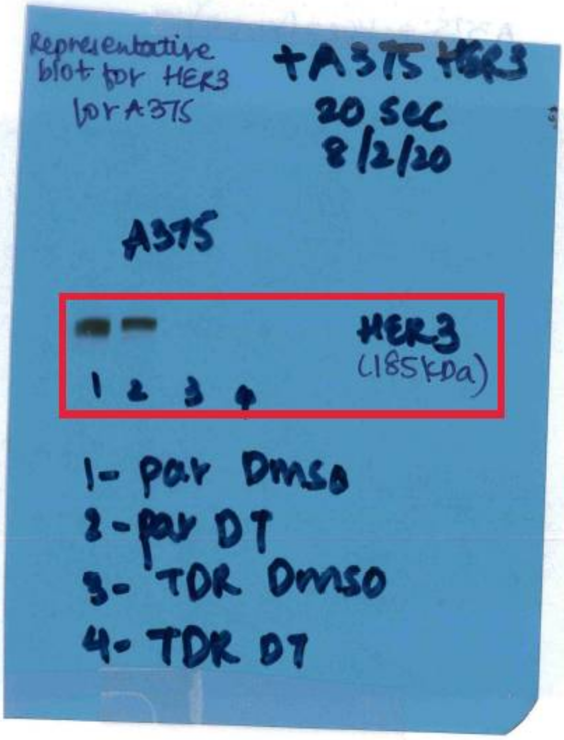

A375pEGFR

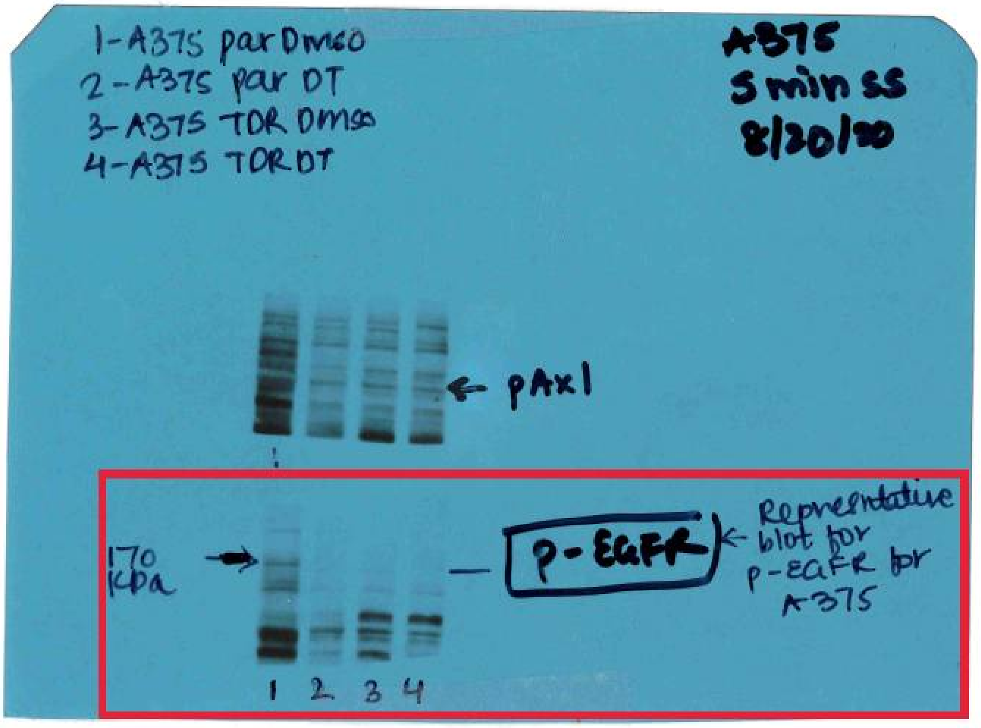

A375: EGFR & IRβ

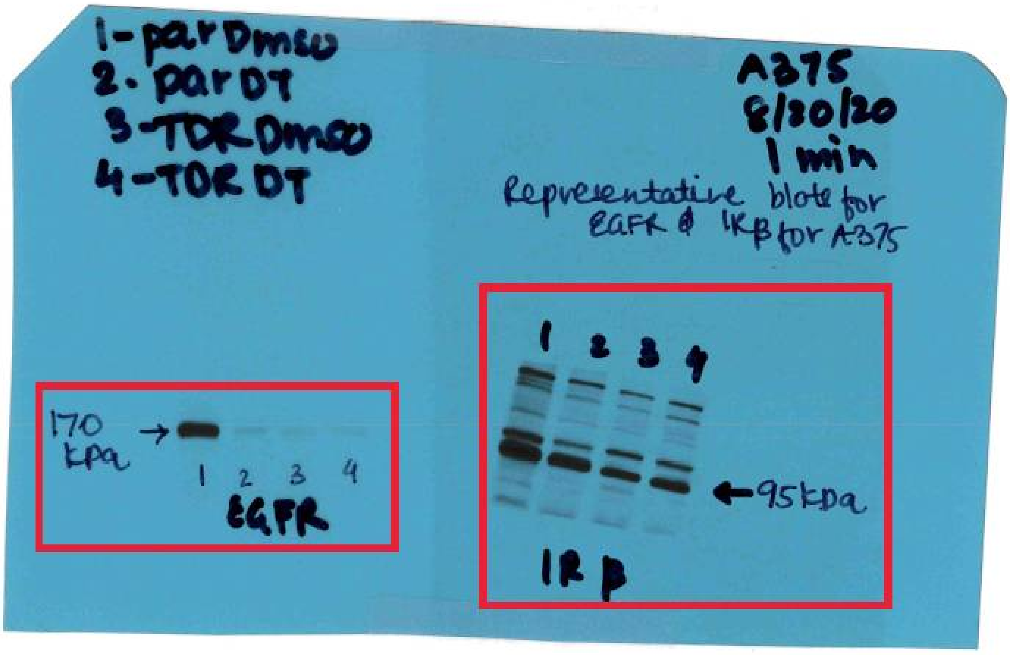

A375 and WM983B p-IGF1R(Y1131) / p-IR(Y1146)

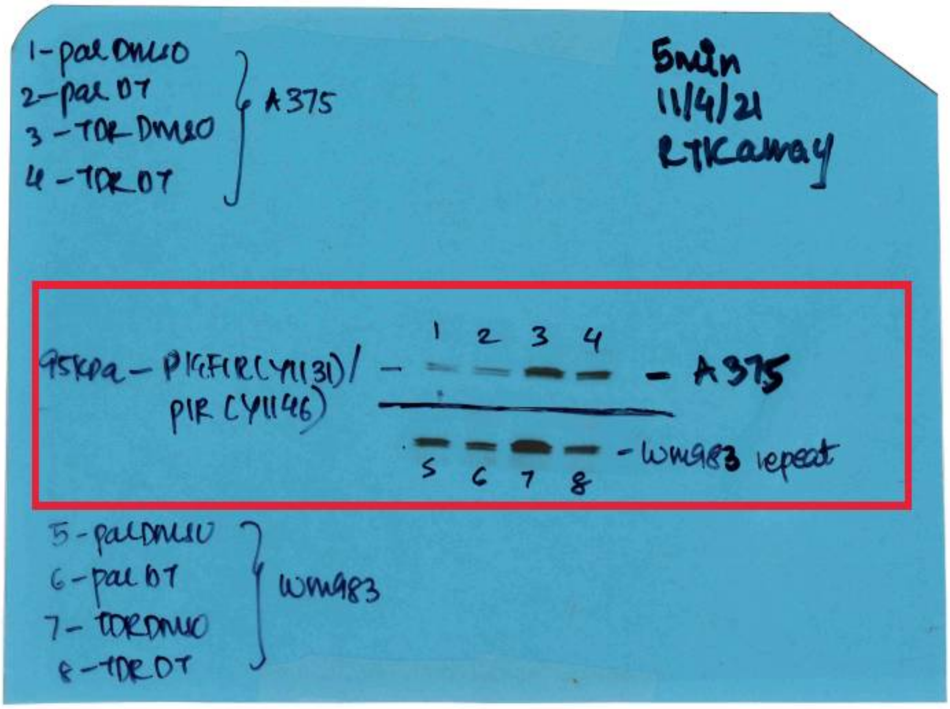

A375: IGF1R

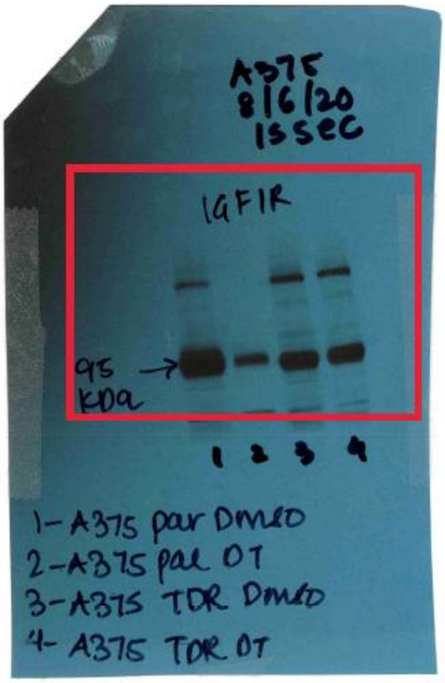

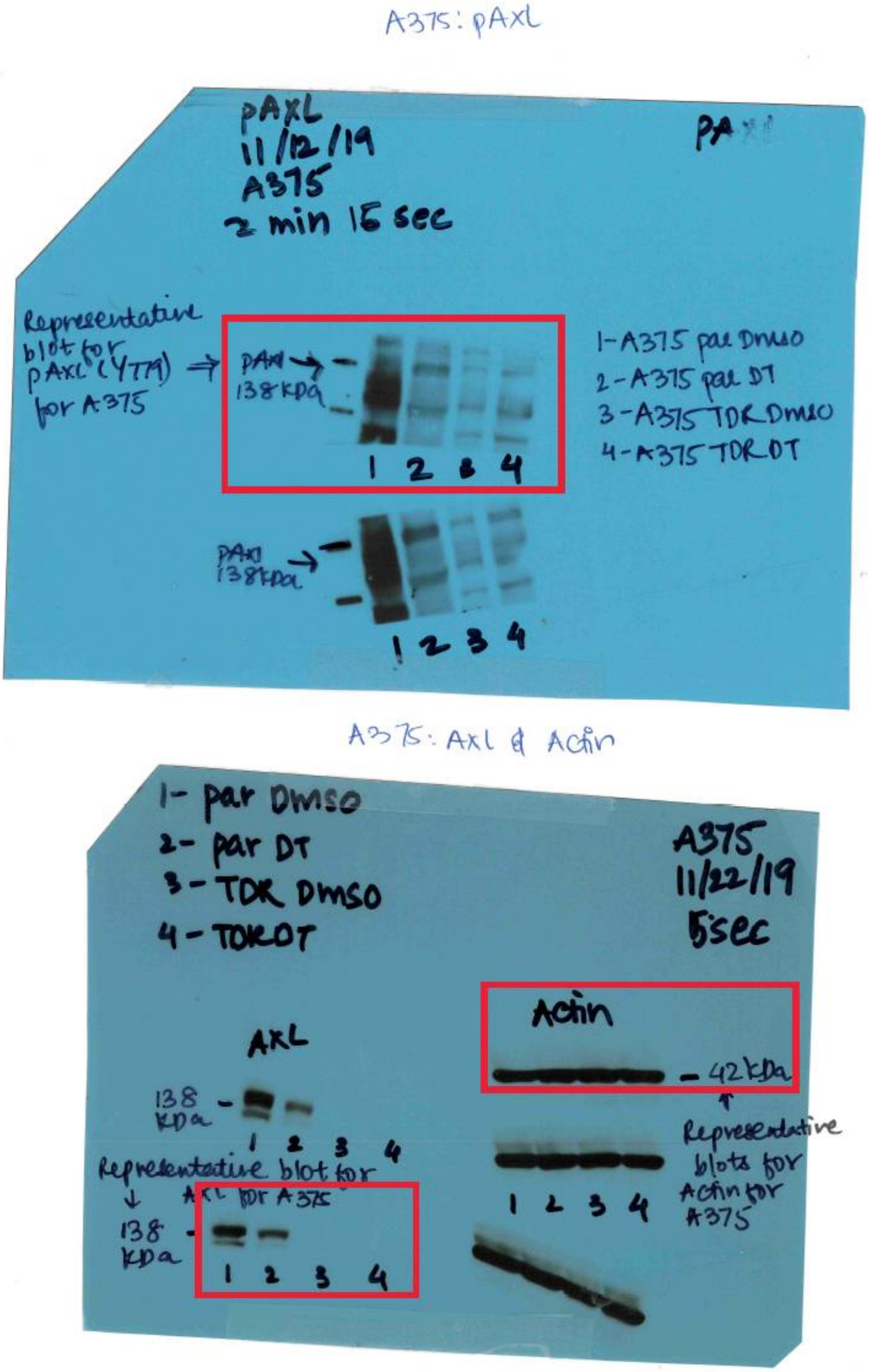

Figure S8. Whole blots for western blots in Figure 2C.

Figure 4D

WM115 TDR: p-IGF1R(Y1131)/pIR(Y1146)

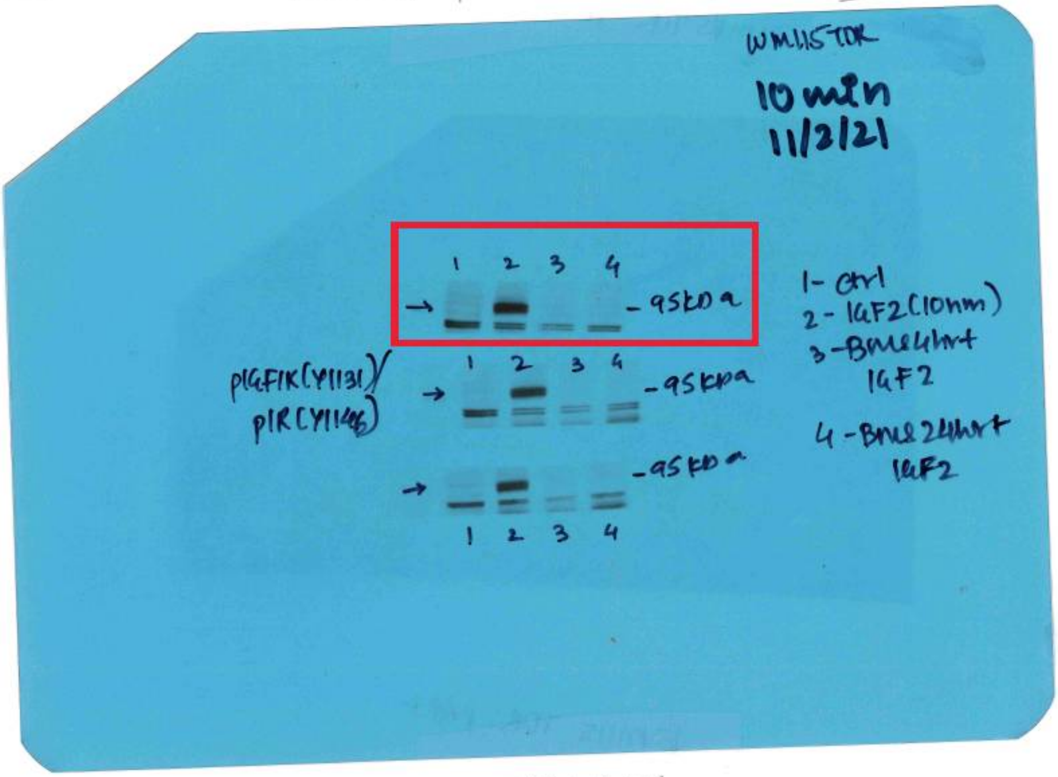

WM115 TDR: IGF1R

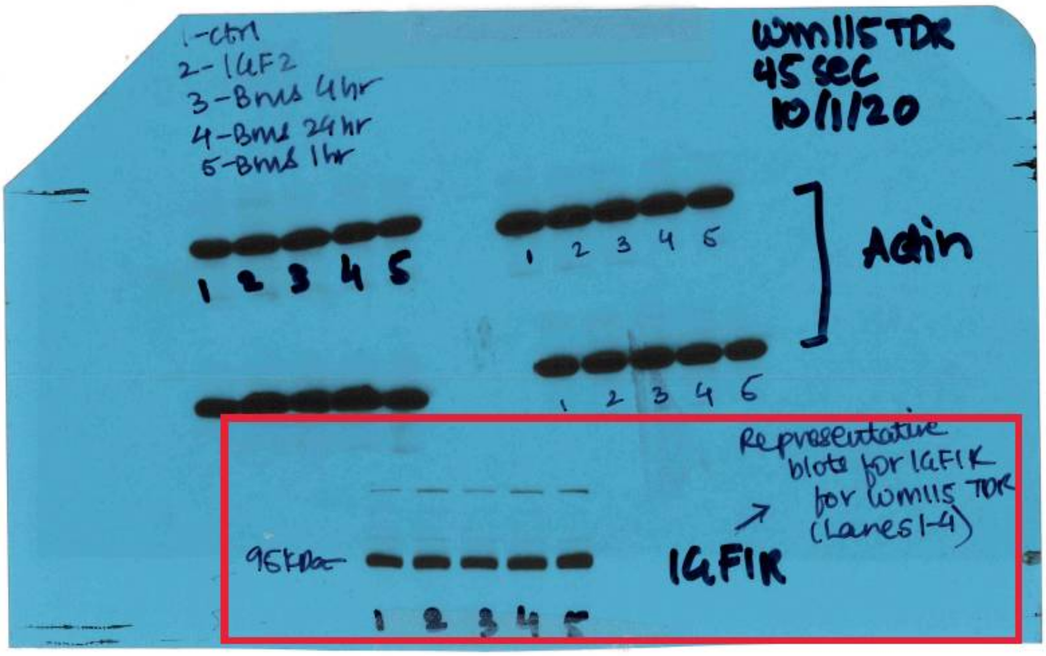

WM115 TDR: IR

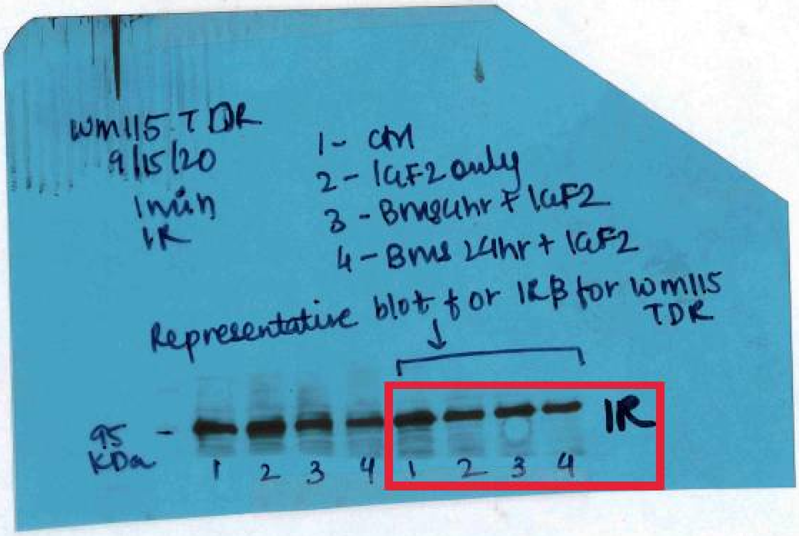

WM115 TDR: pAkt

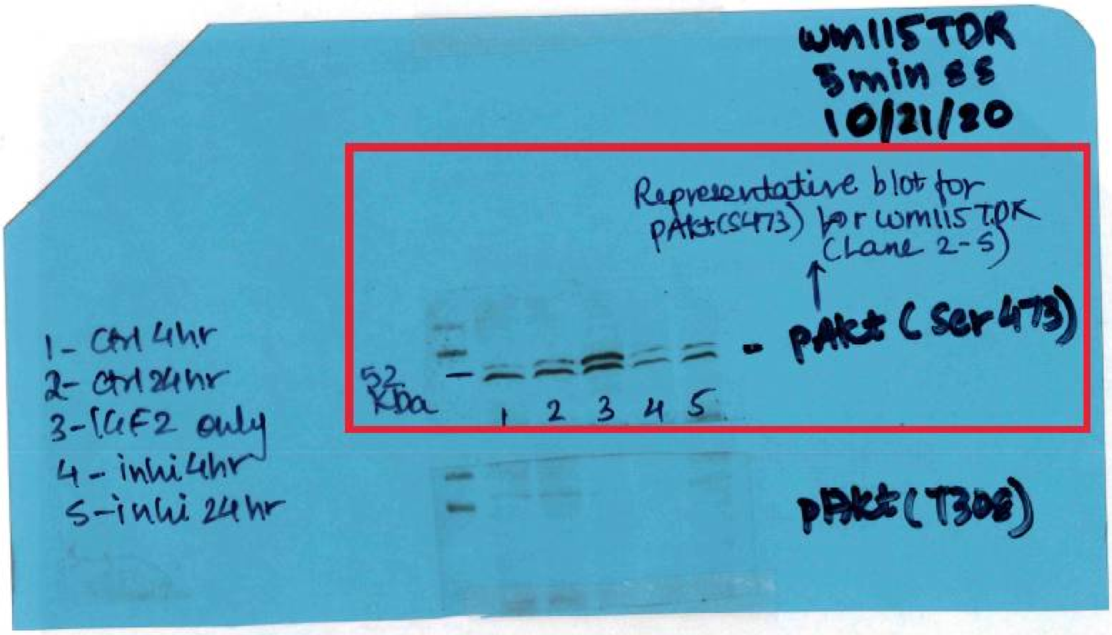

wm115 TDR: ~~[illegible]~~ Akt

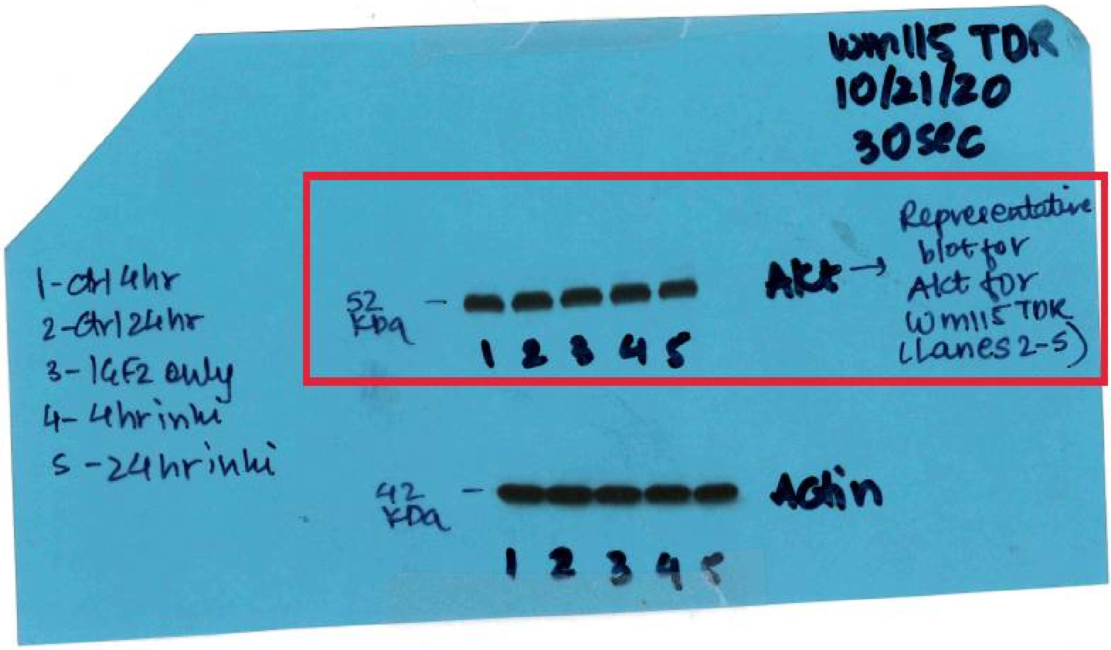

wm115 TDR: p-Erk

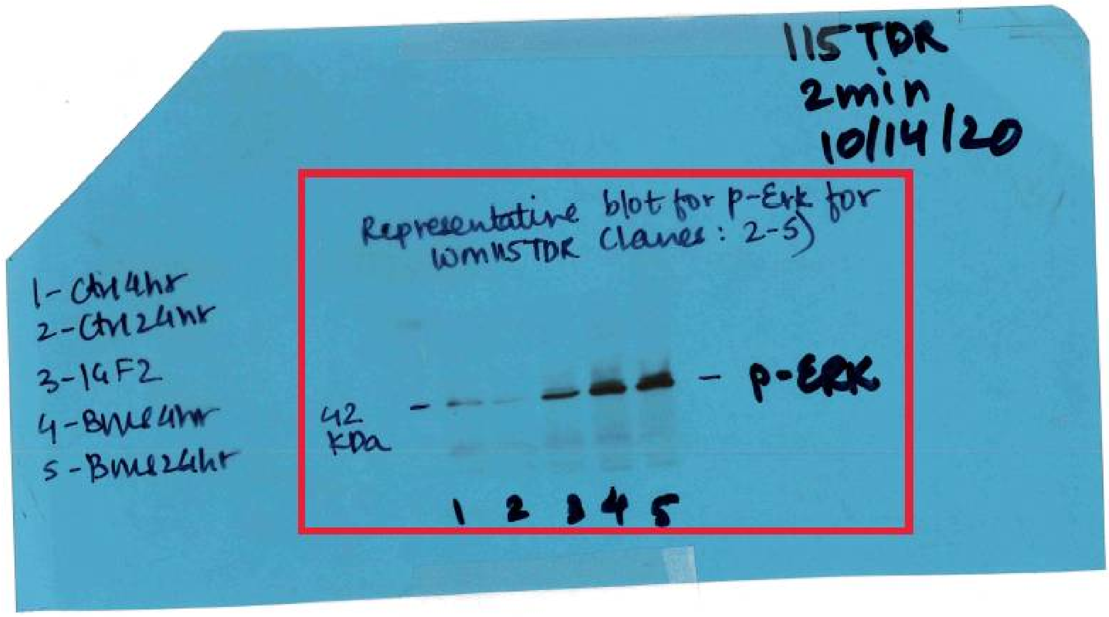

WM115TDR: ERK

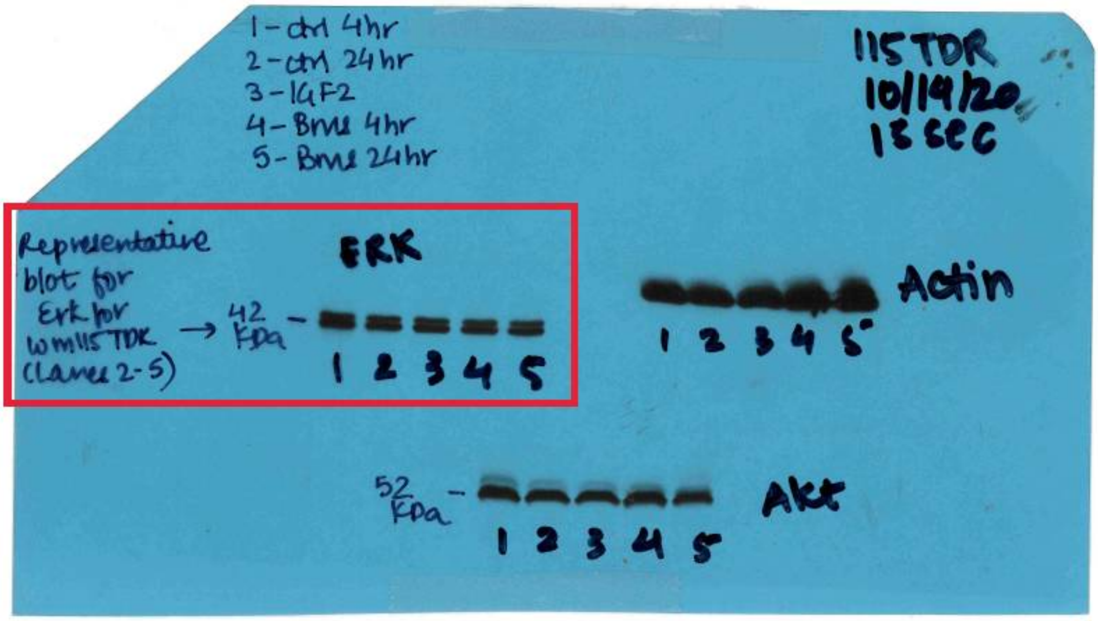

WM115TDR: Actin

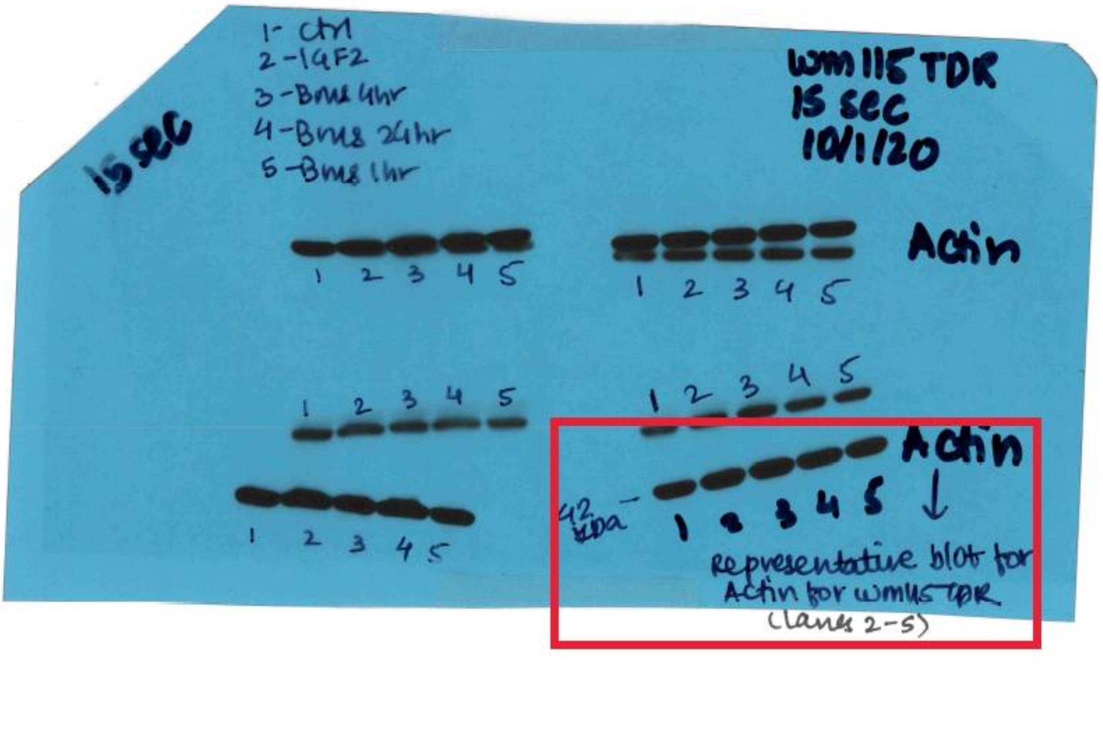

wm983TDR: pIGFIR(Y1131)/pIR(Y1146)

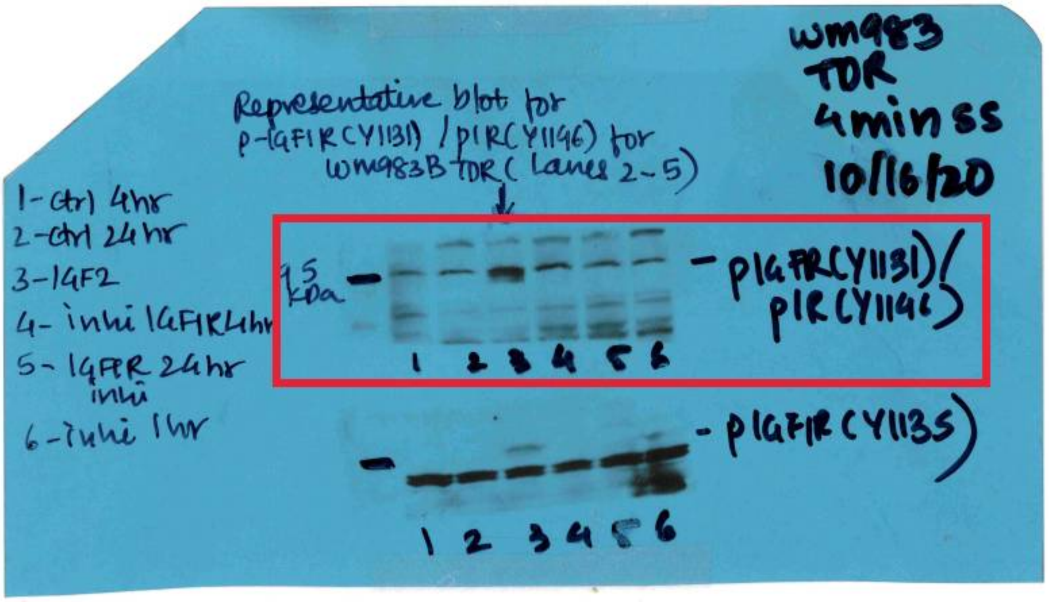

wm983BTDR: IGFIR & IR

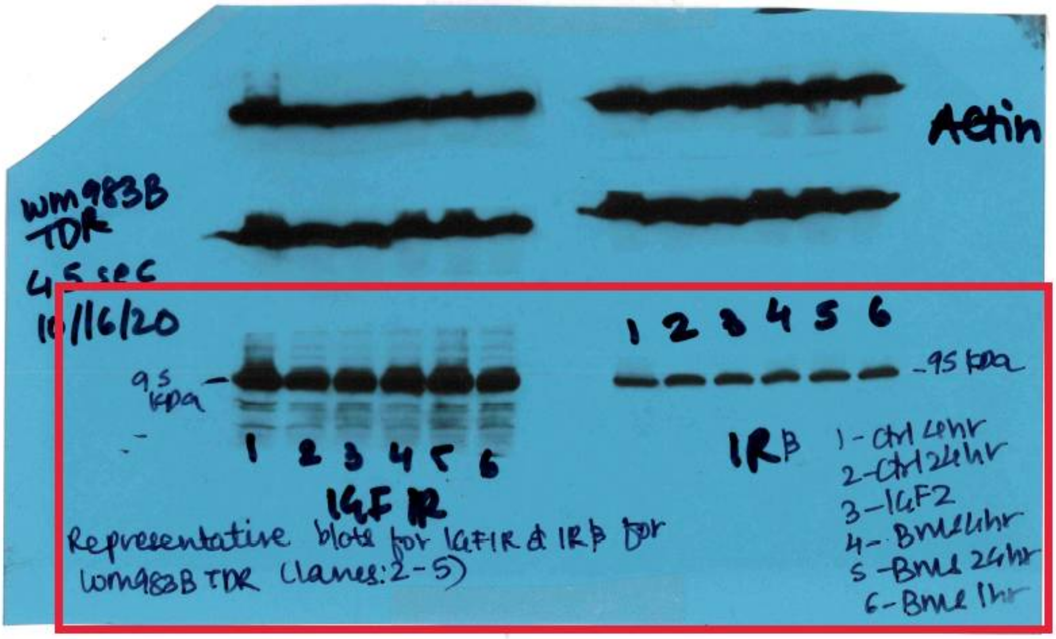

WM983TDR: p-Akt

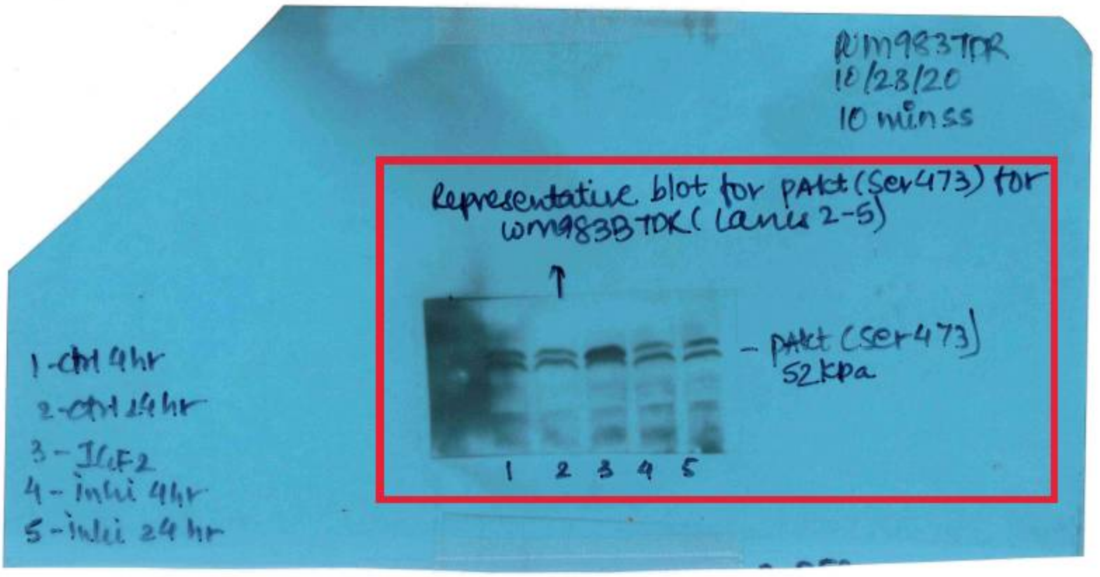

WM983BTDR: AKT

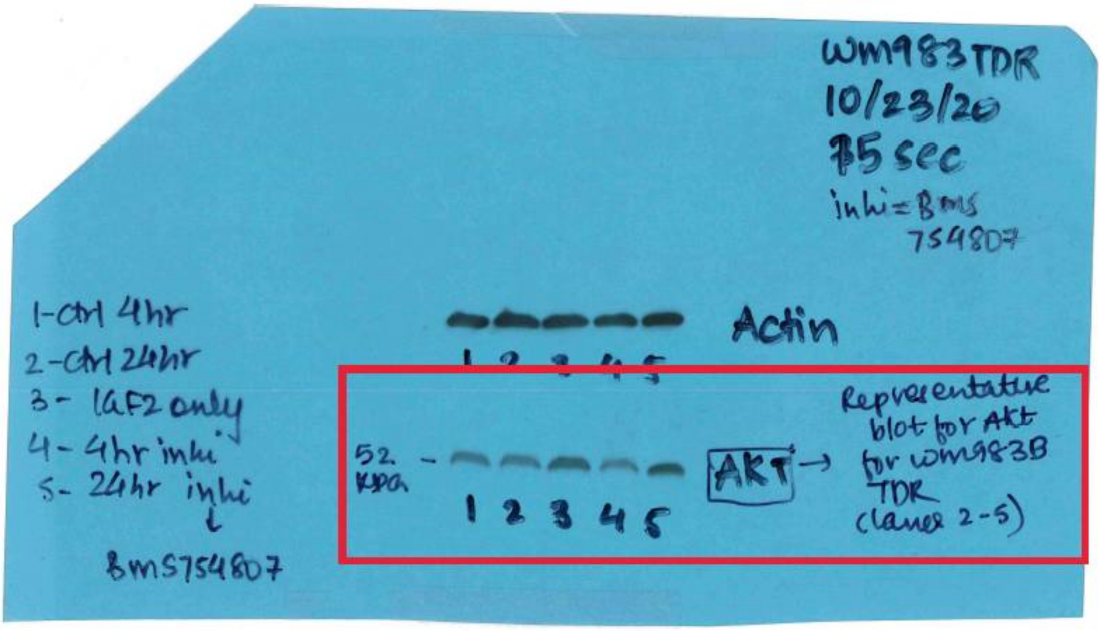

wm983BTDR: P-ERK

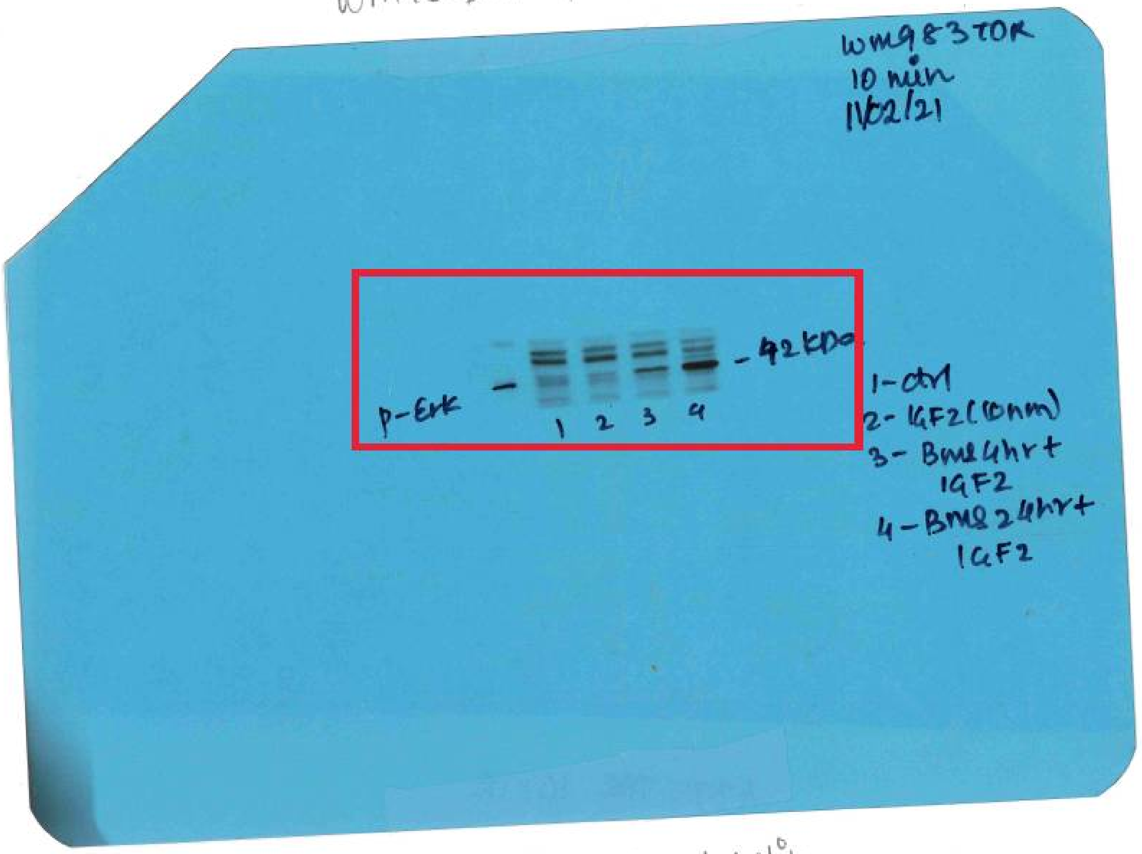

wm983BTDR: ERK & Actin

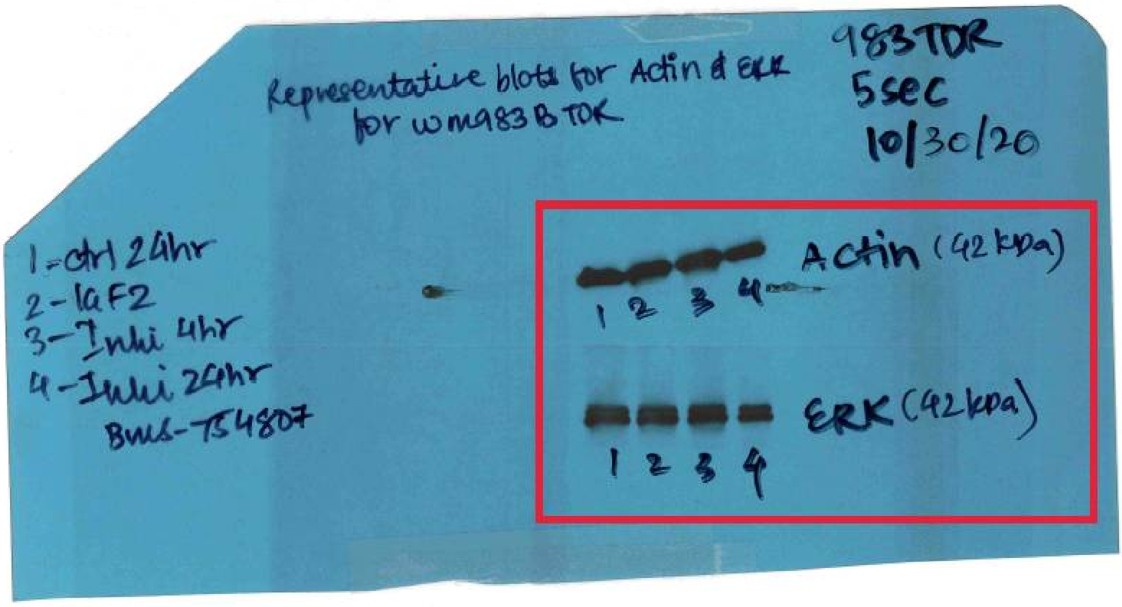

A375TDR: pIGFIR(Y1131)/
pIR(Y1146)

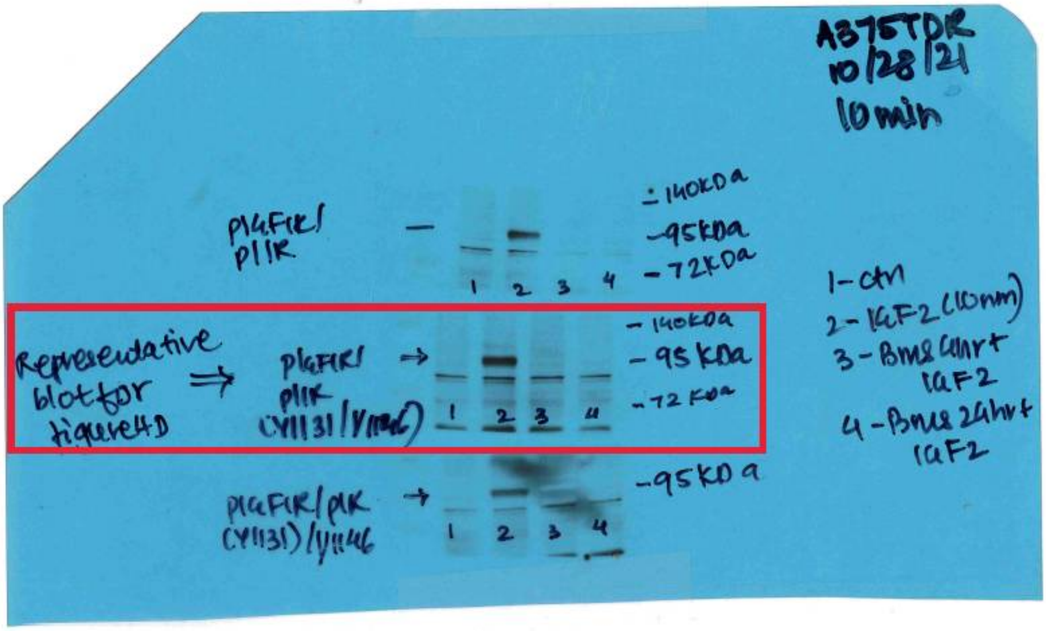

A375TDR: IGFIR

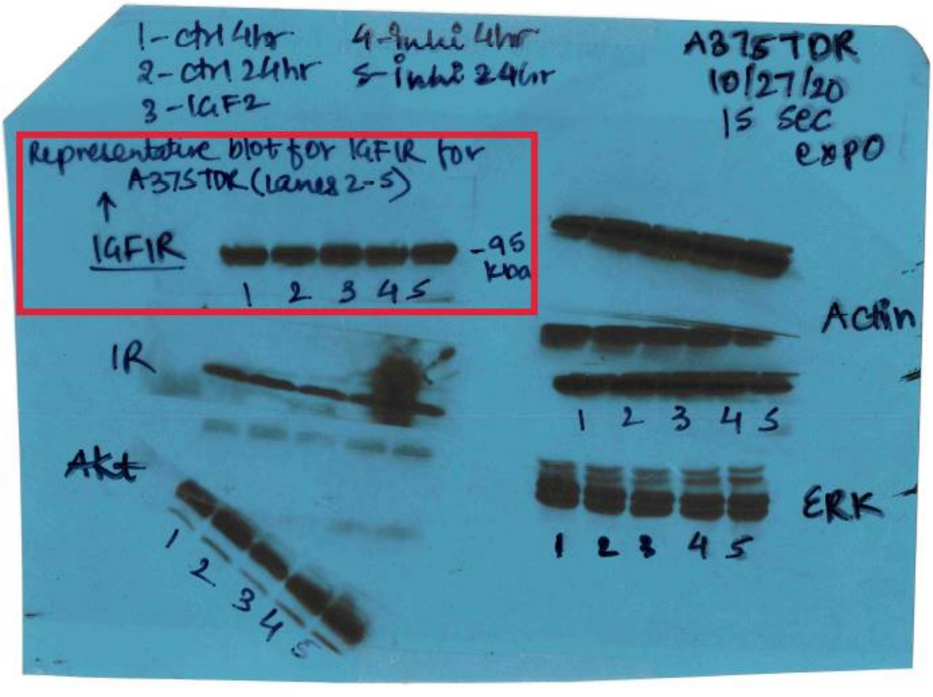

A375TDR: IR

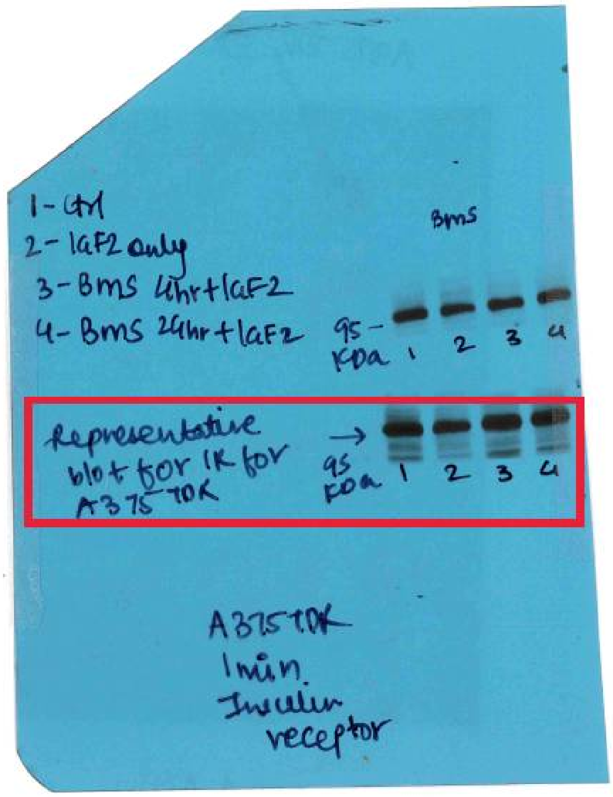

A375 TDR: p-Akt

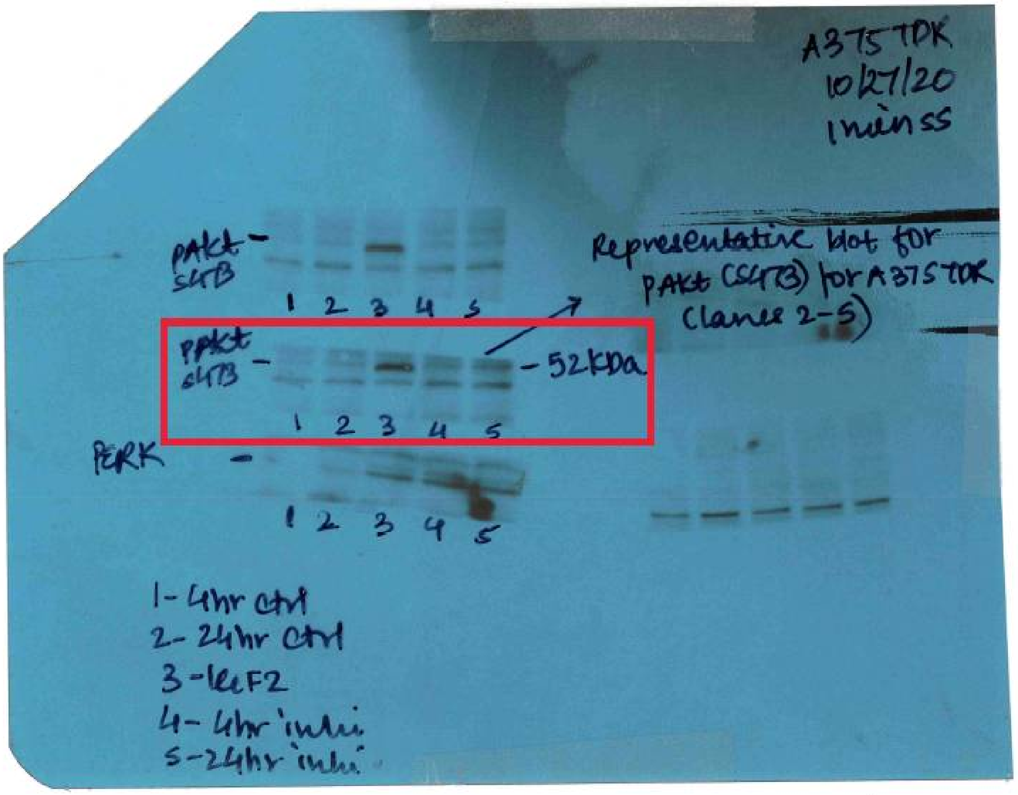

A375 TDR: ERK

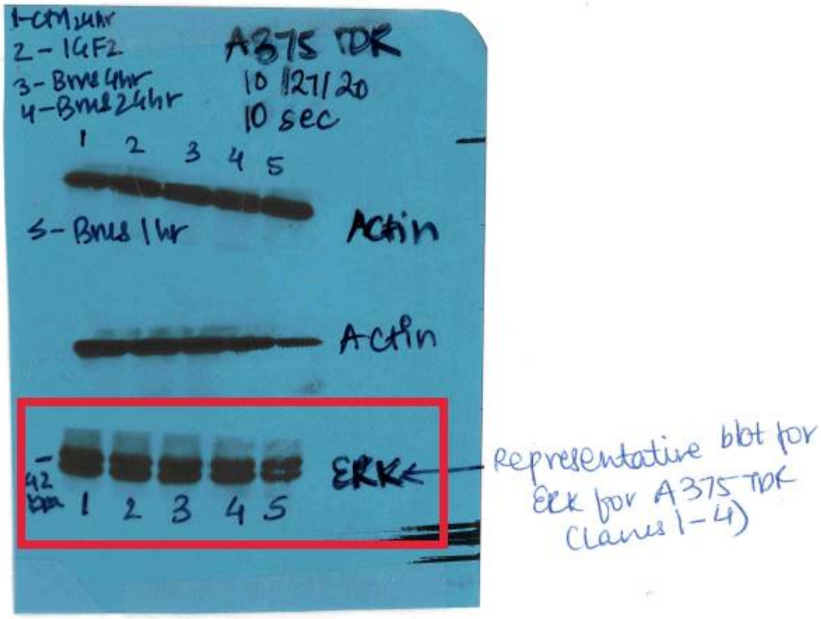

A375 TDR: Akt & Actin

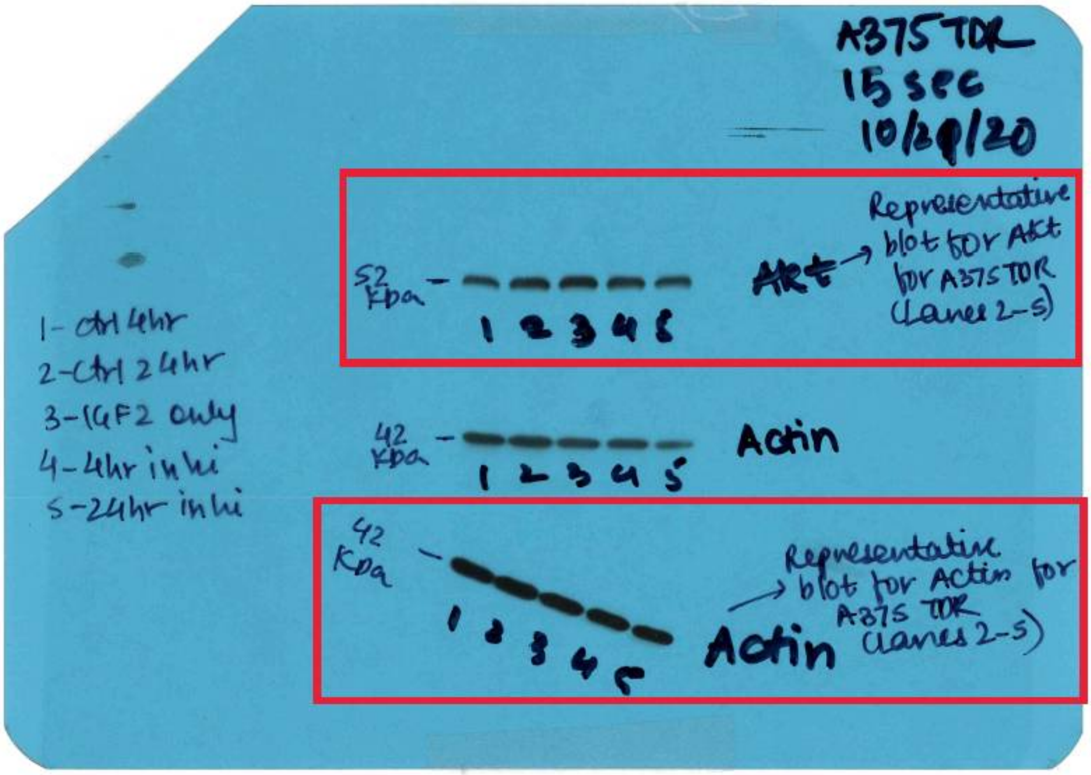

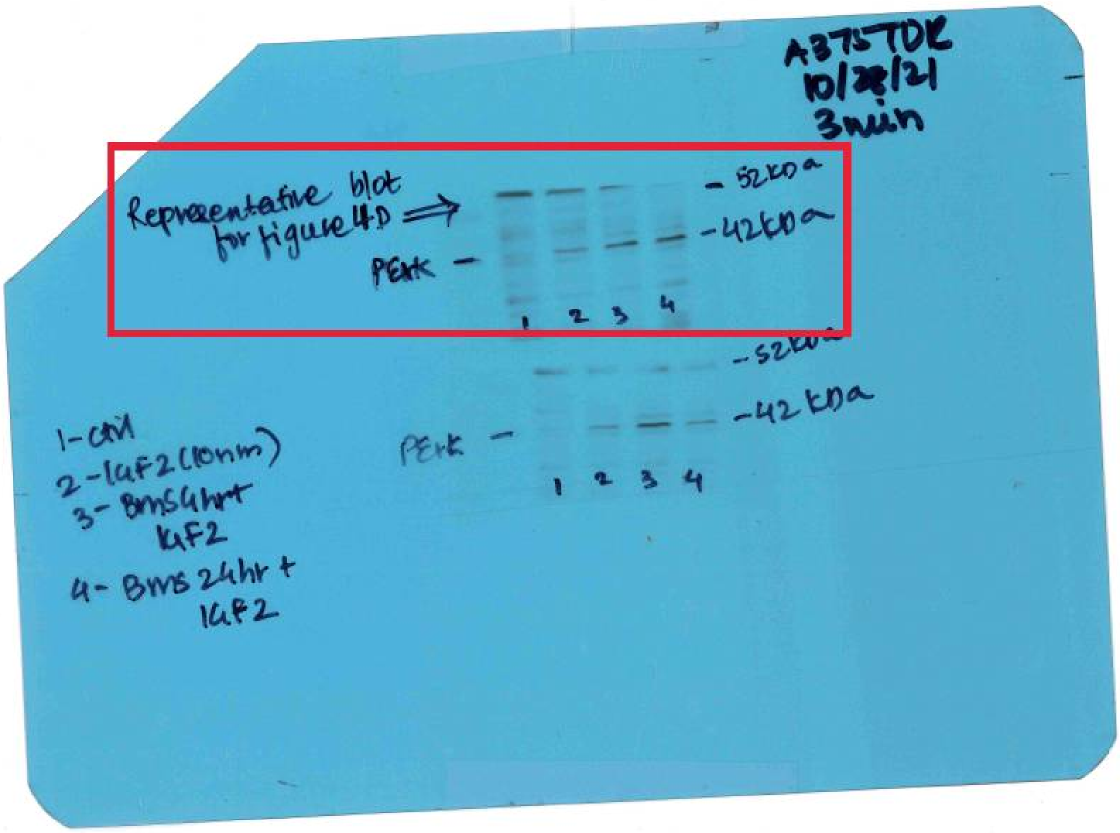

Figure S9. Whole blots for western blots in Figure 4D.

WM115TDR: IGF1R & IR

Representative blots for IGF1R & IR in WM115TDR

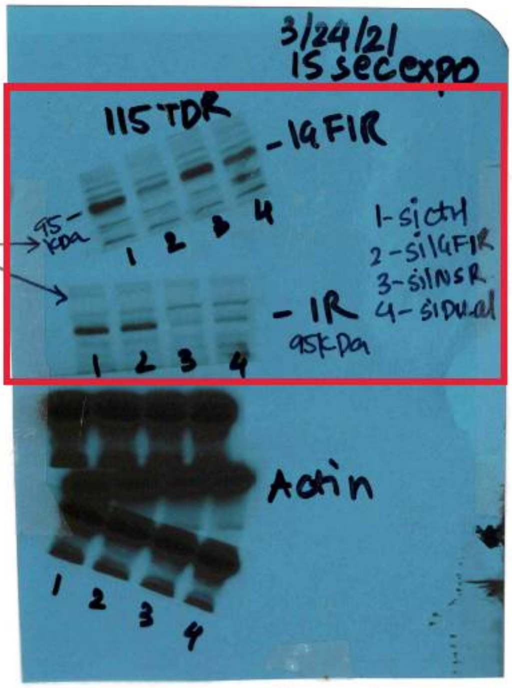

WM115TDR: Actin; WM983BTDR: Actin, IGF1R; A375TDR: IR, Actin

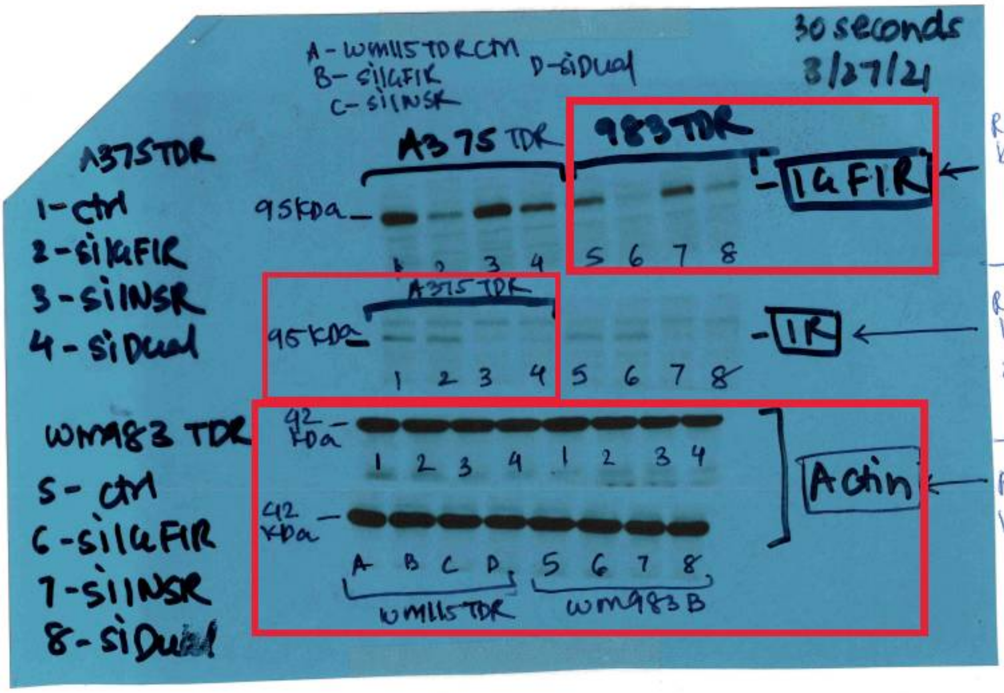

Representative blot for IGF1R for WM983B TDR

Representative blot for IR for A375 TDR

Representative blot for Actin in WM115TDR, WM983B TDR & A375 TDR

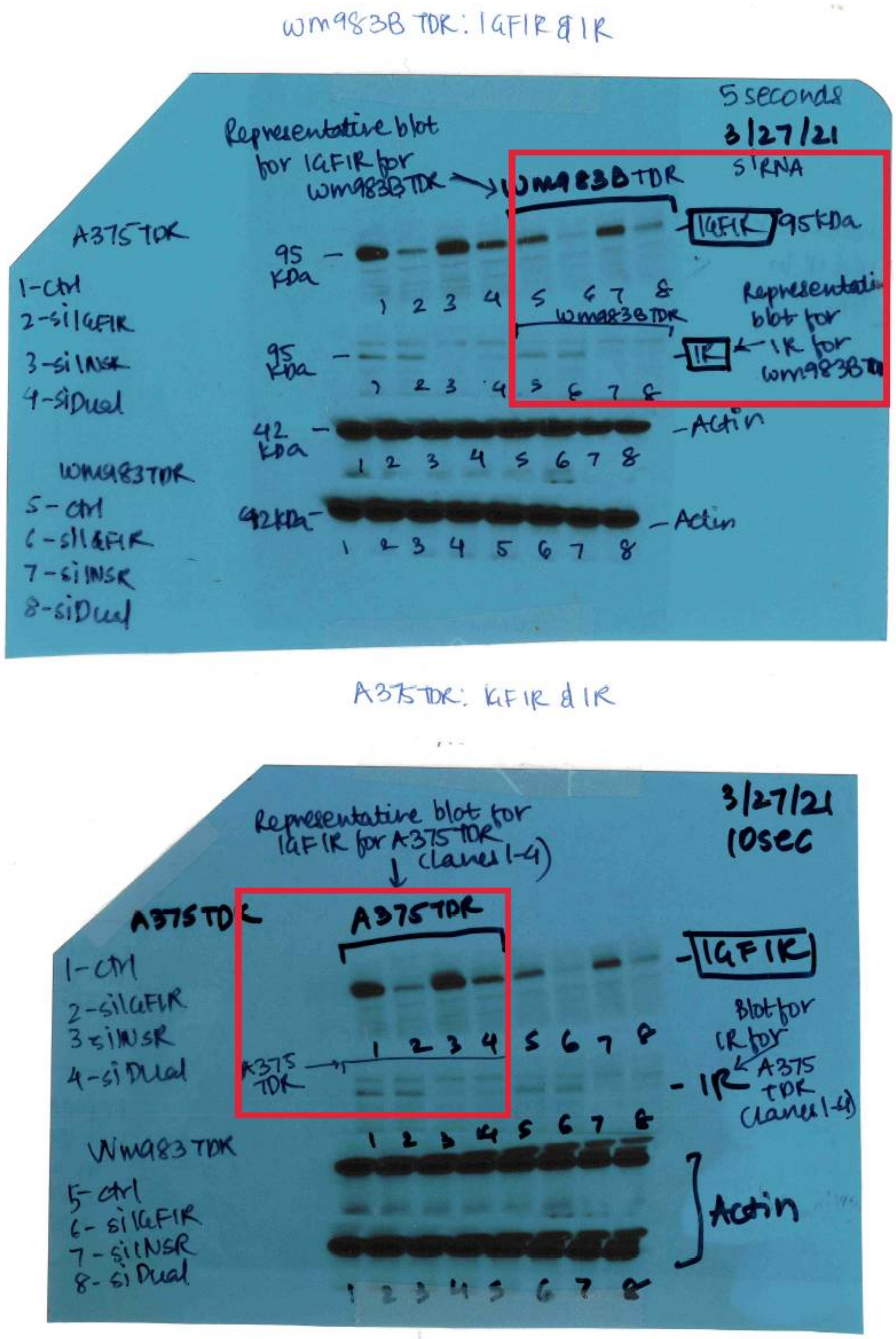

Figure S10. Whole blots for western blots in Figure 5A.

Figure 6
WM115 TDR tumor lysate: p-IGF1R/p-IR

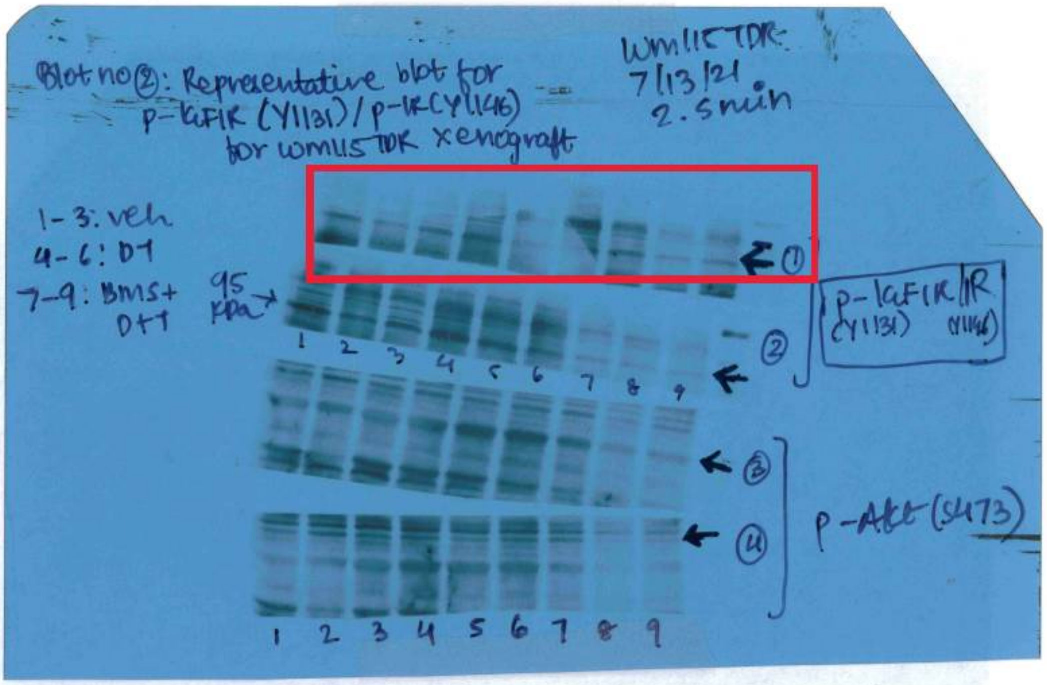

WM115 TDR tumor lysate: p-Akt

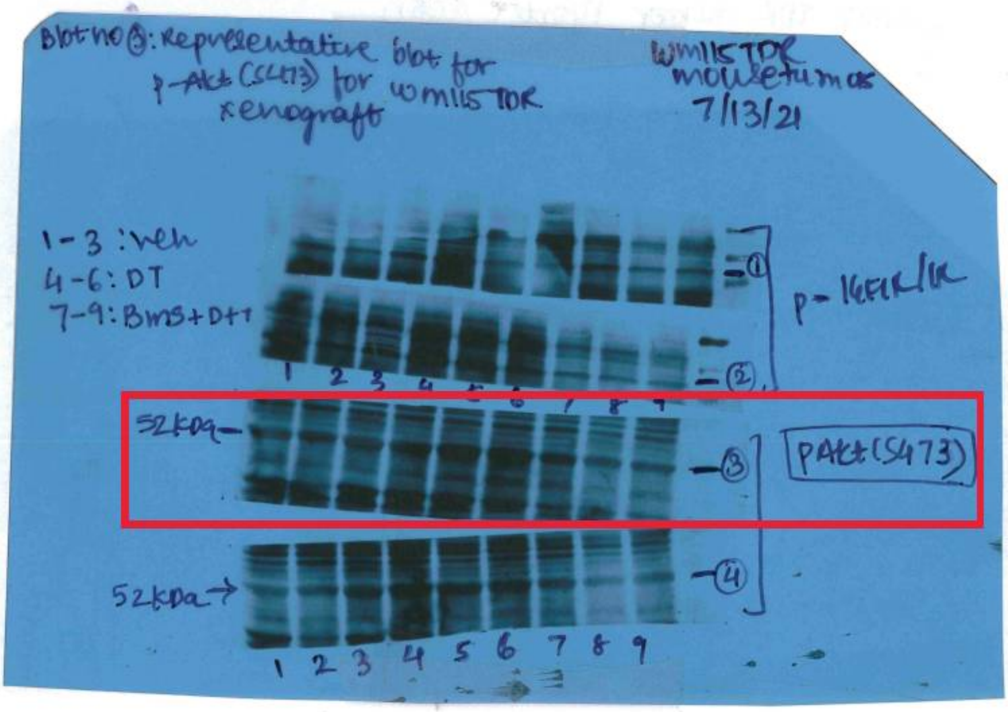

wm115 TDR tumor lysate: IGF1R, IR & Akt

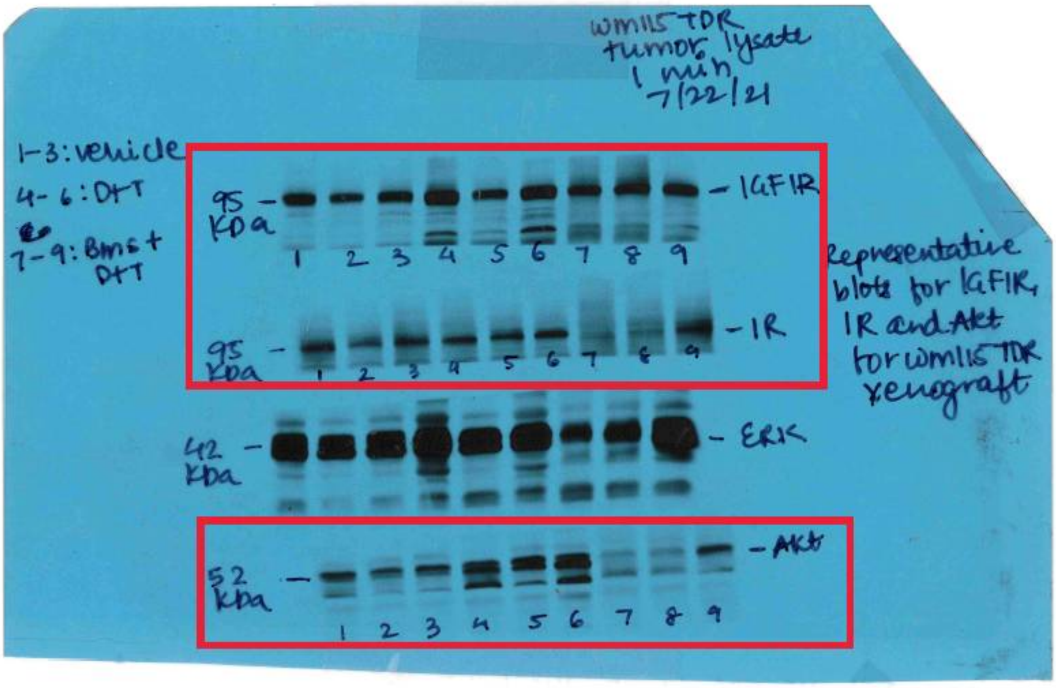

wm115 TDR tumor lysate: Actin

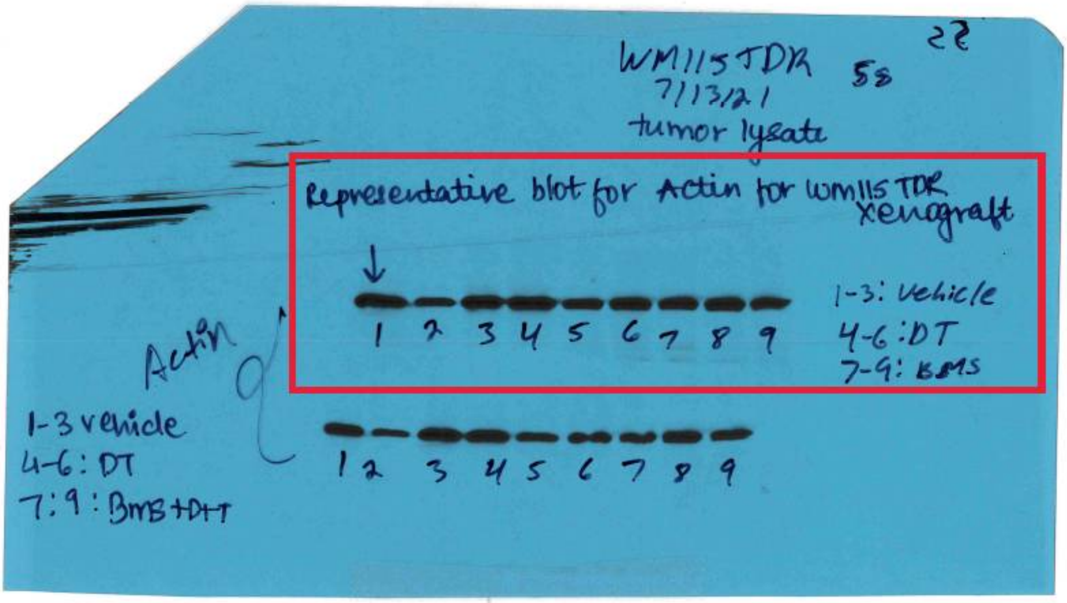

Figure S11. Whole blots for western blots in Figure 6E.

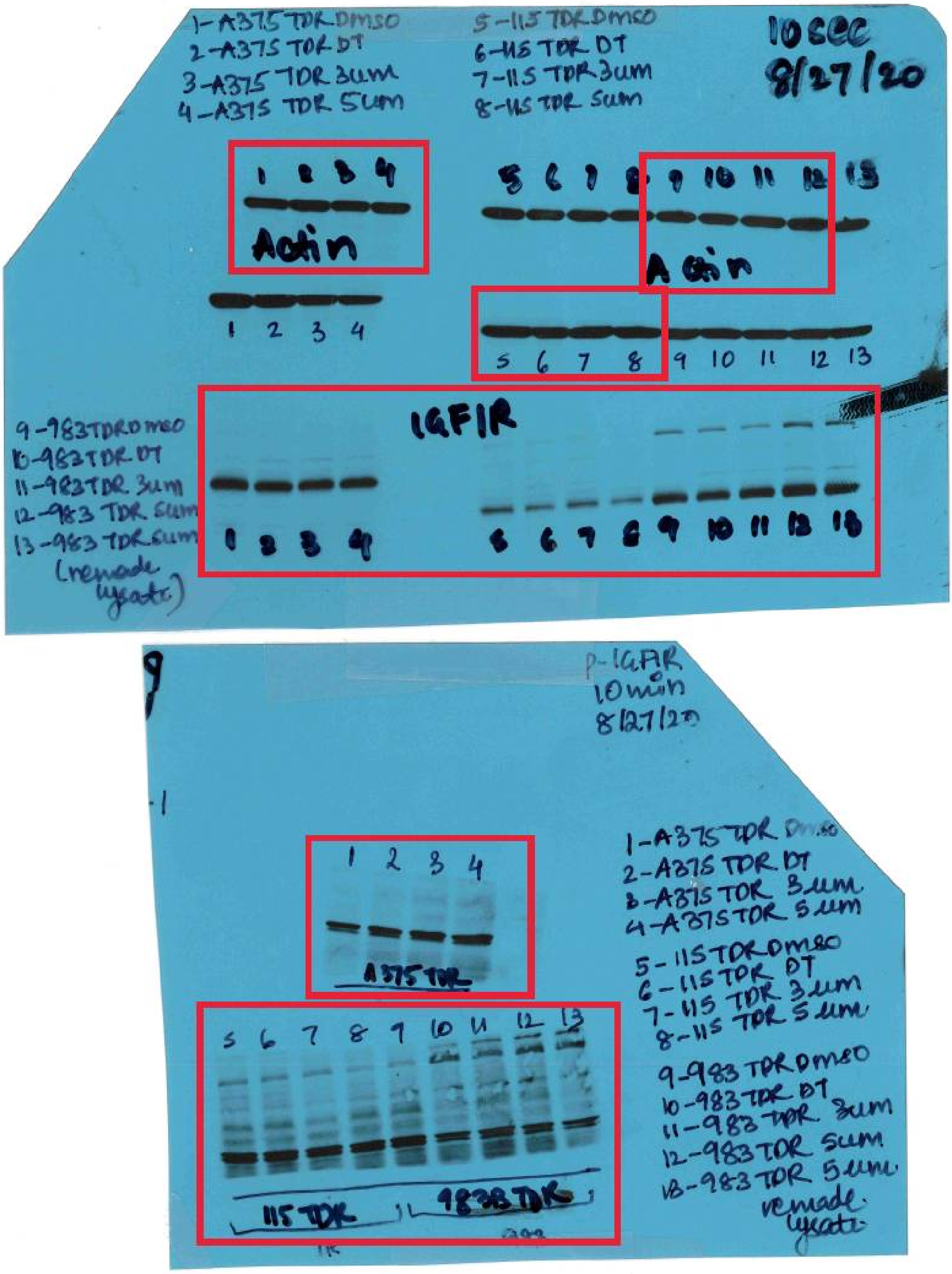

Figure S12. Whole blots for western blots in Figure S4.

Supplementary Figure 4

WM115 TDR: p-IGF1R (Y1135)

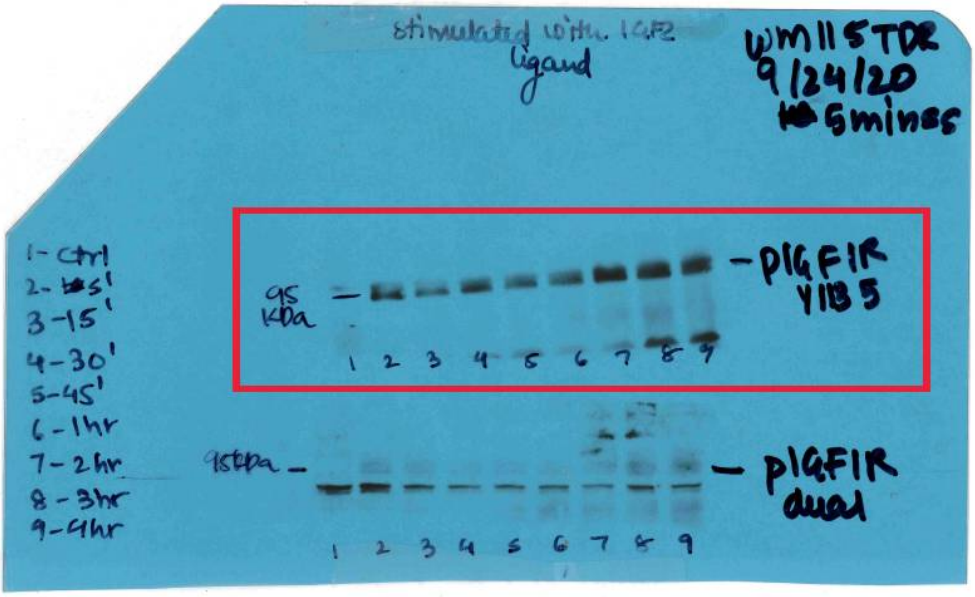

WM115 TDR: P-IGF1R(Y1131) / pIR (Y1146)

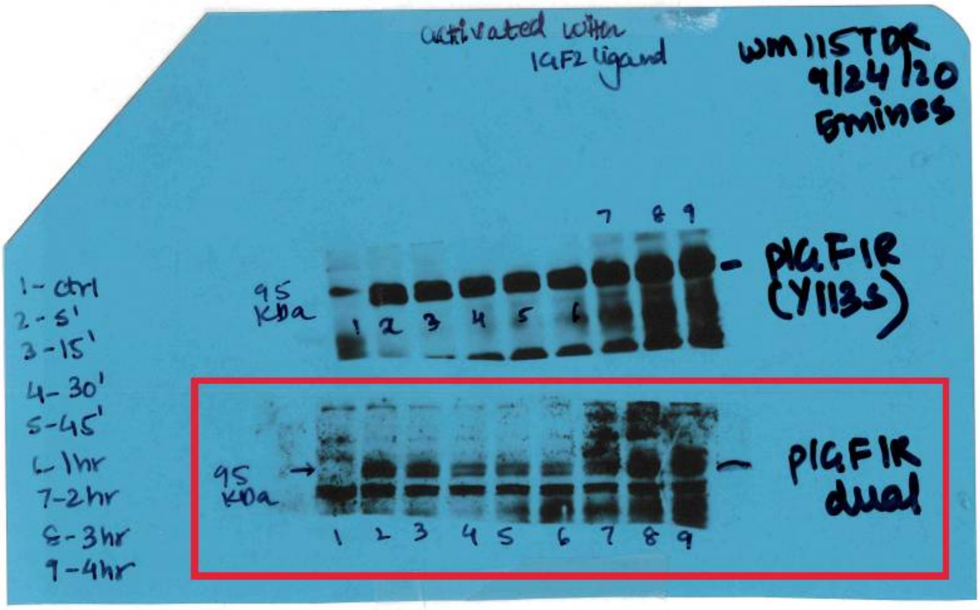

WM115 TDK: IGFIR

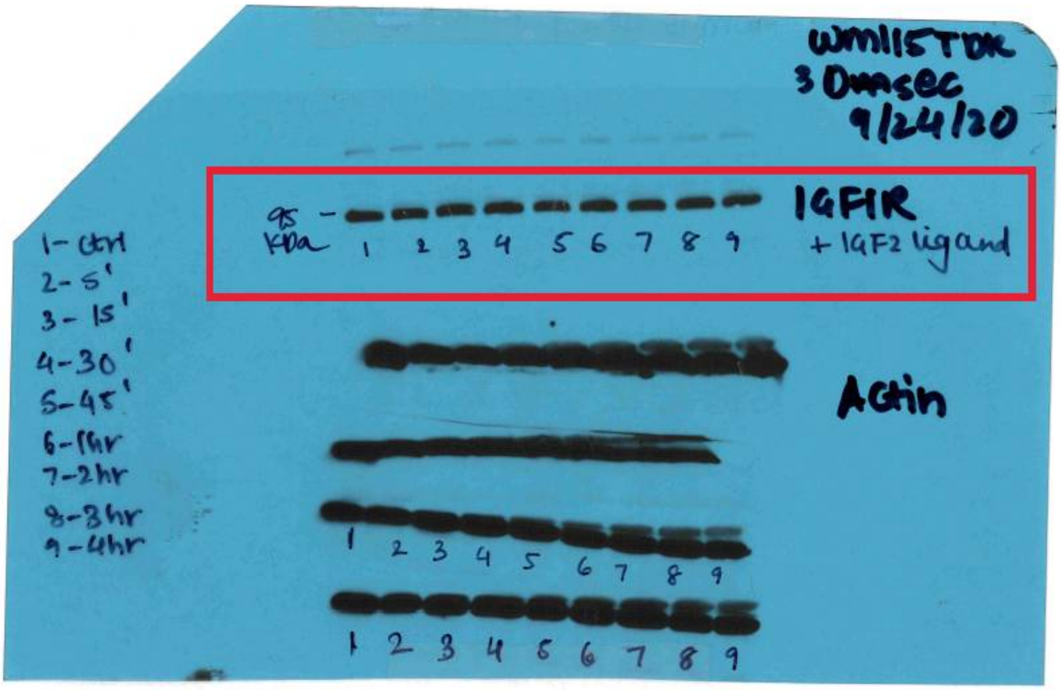

WM115TDR: Actin & IR

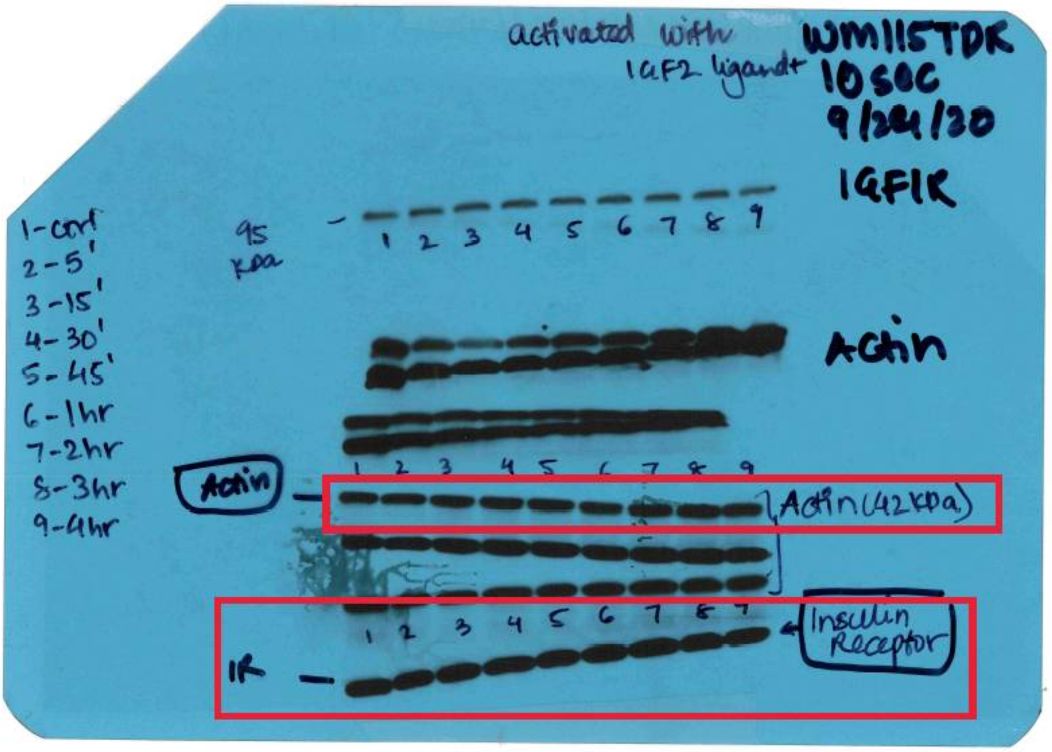

WM983B TDR: p-IGF1R (Y1135)

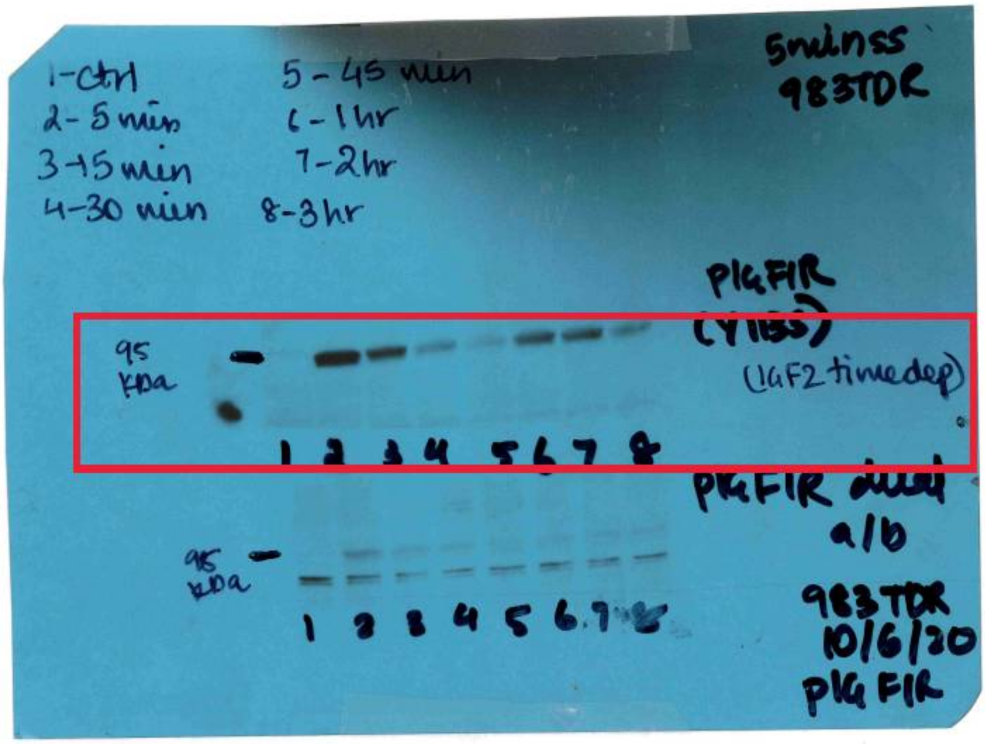

WM983BTDR: P-IGF1R(Y1135/P1R(Y1146)

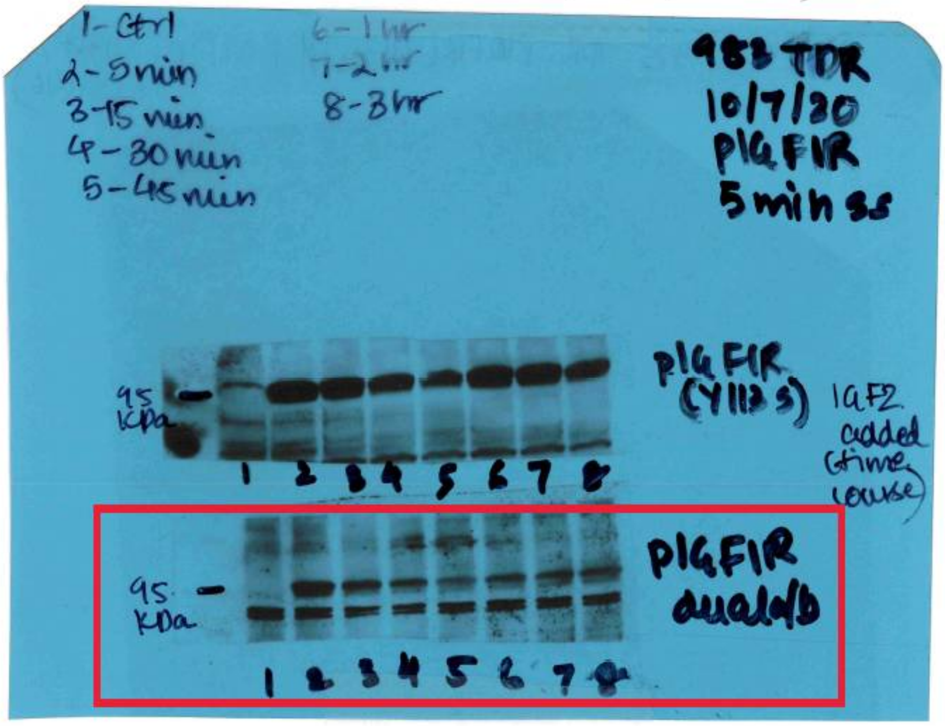

WM983B TDR: IR, IGFIR & Actin

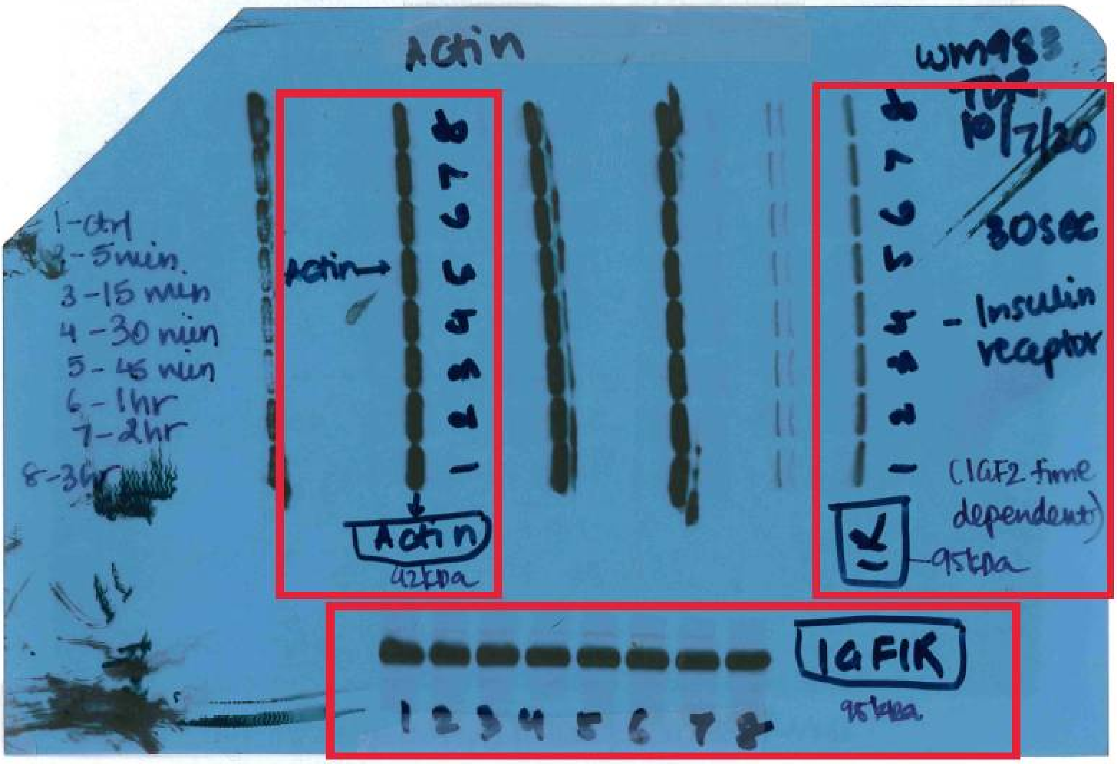

A375 TDR: pIGFIR(Y1135) & p-IGFIR(Y1131) / pIR(Y1146)

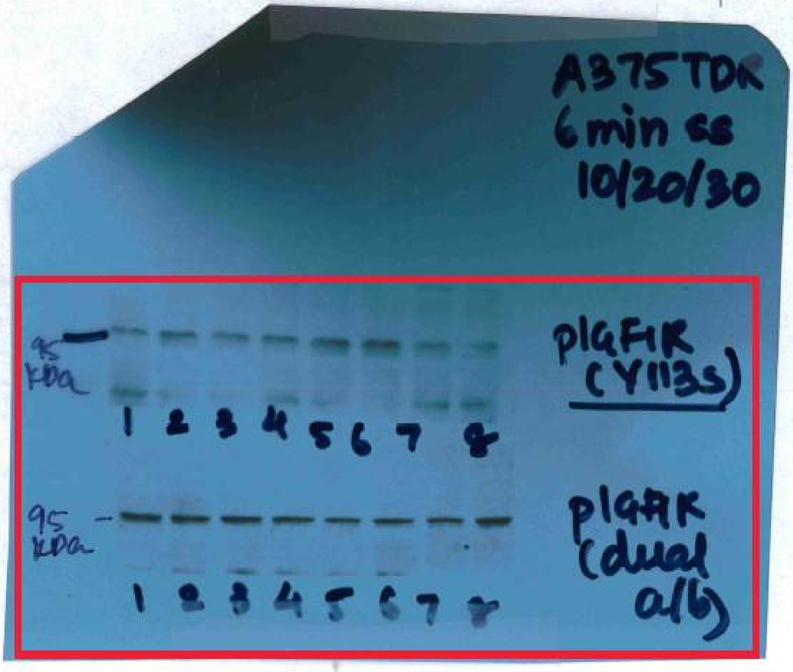

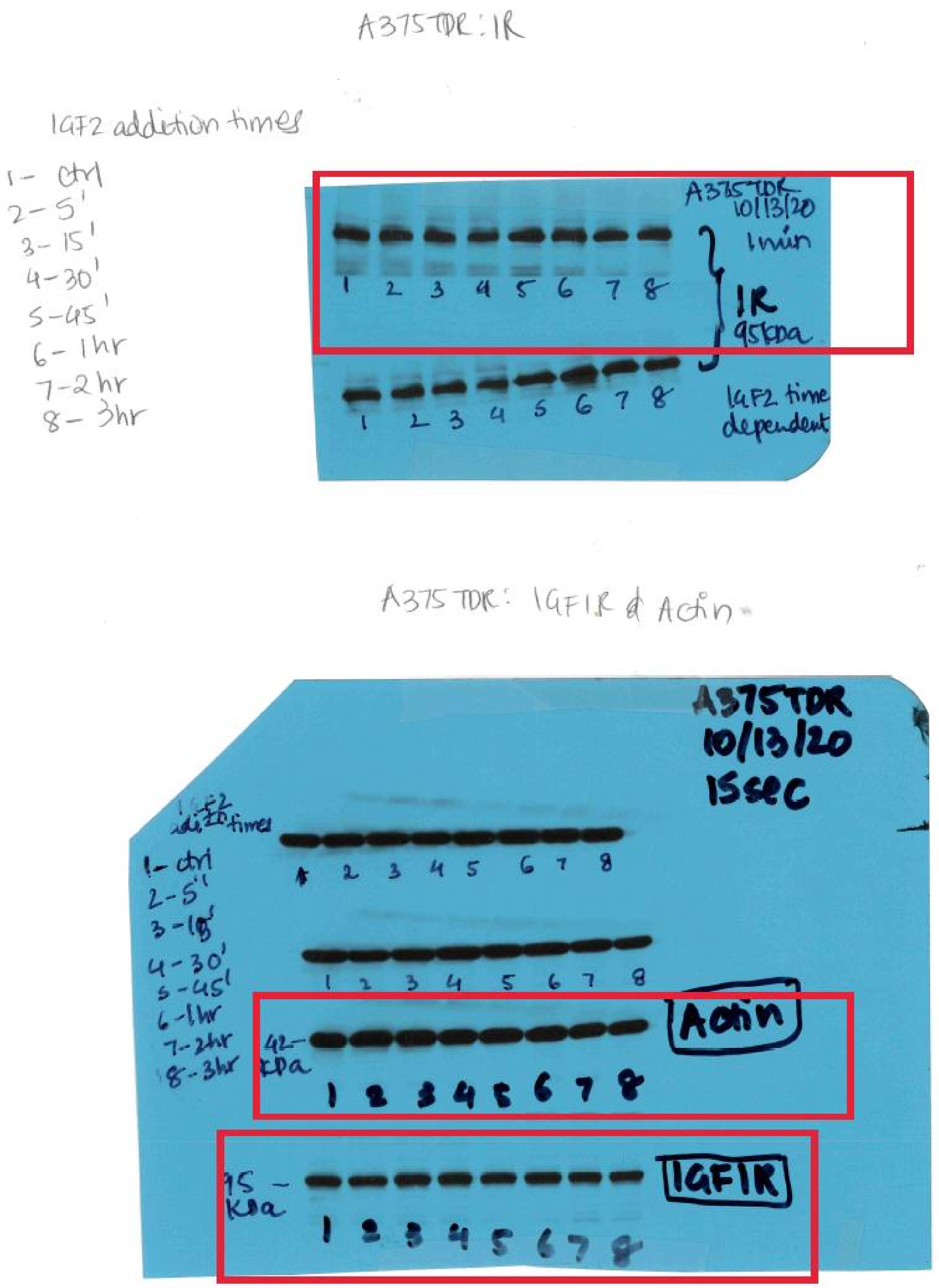

Figure S13. Whole blots for western blots in Figure S5.

Table S1. List of antibodies.

Antibody	Source	Catalog number
Phospho-p44/42 MAPK (Erk1/2) (Thr202/Tyr204)	Cell Signaling Technology	9101
p44/42 MAPK (Erk1/2)	Cell Signaling Technology	9102
β-Actin	Cell Signaling Technology	4970
Phospho-Akt (Ser473)	Cell Signaling Technology	4060
Akt	Cell Signaling Technology	9272
Phospho-HER3/ErbB3 (Tyr1289)	Cell Signaling Technology	4791
Phospho-HER3/ErbB3 (Tyr1197)	Cell Signaling Technology	4561
HER3/ErbB3	Cell Signaling Technology	12708
Phospho-EGF Receptor (Tyr1068)	Cell Signaling Technology	2234
EGF Receptor	Cell Signaling Technology	4267
IGF-I Receptor β	Cell Signaling Technology	3027
Phospho-IGF-I Receptor β (Tyr1135)	Cell Signaling Technology	3918
Insulin Receptor β	Cell Signaling Technology	3025
Phospho-IGF-I Receptor β (Tyr1131)/Insulin Receptor β (Tyr1146)	Cell Signaling Technology	3021
Phospho-Axl (Y779)	R&D Systems	AF2228
Axl	Cell Signaling Technology	8661

Table S2. List of RT-PCR primers.

Gene	Forward Primer Sequence	Reverse Primer Sequence
IGF1R	TCGACATCCGCAACGACTATC	CCAGGGCGTAGTTGTAGAAGAG
IGF1	TCGGTAACTGACTTGAATGTCCA	TCCTTTTTCGCTTCCCTGTTTT
IGF2	GGGCAAGTTCTTCCAATATGA	TCACTTCCGATTGCTGGC
INSR	AAAACGAGGCCCGAAGATTTC	GAGCCCATAGACCCGGAAG
NRG1	GCTTCATGGTGAAAGACCTTTCA	ATTACGTAGTTTTGGCAGCGATC
HER3	GGTGATGGGGAACCTTGAGAT	CTGTCACTTCTCGAATCCACTG
AXL	GTGGGCAACCCAGGGAATATC	GTACTGTCCCGTGTCGGAAAG
GAS6	GGTAGCTGAGTTTGACTTCCG	GACAGCATCCCTGTTGACCTT
PROTEIN S	TCCTGGTTAGGAAGCGTCGT	CCGTTTCCGGGTCATTTTCAAA
ACTIN	AAGGAGCCCCACGAGAAAAAT	ACCGAACTTGCATTGATTCCAG
EGFR	AGCTAACCCATTATGGCAACA	AGTTTTCACTGAGTCAGCTCCAT
HBEGF	GAAAGACTTCCATCTAGTCACAAAGA	GGGAGGCCCAATCCTAGA
TGFA	GGACAGCACTGCCAGAGA	CAGGTGATTACAGGCCAAGTAG
BETACELLULIN	TGCCCCAAGCAATACAAGC	CGTCTGCTCGGCCACC
AMPHIREGULIN	ATATCACATTGGAGTCACTGCCCA	GGGTCCATTGTCTTATGATCCAC
